# Supplementary material for: Nutrigenetics-based intervention approach for adults with non-alcoholic fatty liver disease (NAFLD): study protocol for a randomised controlled feasibility trial
Source: BMJ Open. 2021 Apr 8;11(4):e045922. doi: 10.1136/bmjopen-2020-045922 (PMC8039279; doi:10.1136/bmjopen-2020-045922)
Supplement: Supplementary data [file bmjopen-2020-045922supp001.pdf]

Appendix

Table 1. Mediterranean Diet Meal Options

Meal (one serving per container)	Protein(g)	Carb(g)	Fat(g)	Calories
Beef Bolognese with vegetables and whole-wheat pasta	42	43	14	466
Romesco chicken with vegetables and whole-wheat pasta	38	45	17	485
Garlic and herb chicken with vegetable medley	36	27	16	396
Chicken, basil and tomato stir fry	36	40	18	466
Mediterranean falafels with vegetable medley and tahini	10	39	15	331
Cod on vegetable pasta with tomato and basil sauce	30	45	14	426
Halloumi cashew nut and broccoli curry	26	50	23	511
Tomato, black bean with cashews and quinoa	12	36	16	361
Tomato chickpea vegetable curry	14	38	13	353
Chipotle sweet potato chilli with spinach	20	65	6	394
Chicken salad with pesto pasta	36	30	15	399
Garlic and herb chicken salad	32	25	9	309
Tuna and cheese with pesto pasta salad	36	38	12	404
Feta, pine nut and pesto cous salad	16	40	17	377
Red pesto salmon with vegetable medley	24	40	26	490
Honey chilli salmon salad	23	25	20	313
Mozzarella, roasted tomato and pesto pasta salad	19	47	25	477
Grilled halloumi and roasted pepper salad	23	25	23	399
Falafel salad with tahini, avocado and sriracha in Mediterranean herb wrap	20	55	18	462
Chicken with broccoli cashew nut pesto pasta	38	39	17	461
Lentil dahl	23	75	5	437
Vegetable balls and tomato pesto sauce and pasta	12	56	16	416
Chickpea and vegetable curry with rice	23	70	16	516
Butternut, lentil and spinach	15	28	18	372
