# Supplementary material for: Nutrigenetics-based intervention approach for adults with non-alcoholic fatty liver disease (NAFLD): study protocol for a randomised controlled feasibility trial
Source: BMJ Open. 2021 Apr 8;11(4):e045922. doi: 10.1136/bmjopen-2020-045922 (PMC8039279; doi:10.1136/bmjopen-2020-045922)
Supplement: Supplementary data [file bmjopen-2020-045922supp002.pdf]

Appendix 2: Guide for open-response questions

1. How do you feel about the number and duration of the visits and telephone contacts delivered in the trial?

2. What are your thoughts on the main tests we asked you to complete?
   prompts
   - weight, waist, hip and body composition measures
   - blood pressure
   - questionnaires
   - accelerometer
   - urine samples
   - blood samples
   - web-based diet assessment tool

3. Did you complete the main tests and follow the diets as planned? If not, what were the reasons for this?

4. Are there any aspects of the content or delivery of the trial you think we could improve upon?

5. How did your diet and lifestyle patterns change during COVID-19?

The following questions relate to the foods supplied from the Mediterranean diet supplier.

6. Can you tell me your thoughts on the range of foods available?

7. What is your opinion on the taste of the foods that you selected?

8. Can you describe how appealing or unappealing you found the appearance of the selected foods?

9. Can you share your thoughts on how easy, or difficult, was it to use the company's website to order foods?

10. What is your opinion of the delivery times?

11. Do you have any other comments about the Mediterranean meal provider you would like to share with us?
