# Supplementary material for: Nutrigenetics-based intervention approach for adults with non-alcoholic fatty liver disease (NAFLD): study protocol for a randomised controlled feasibility trial
Source: BMJ Open. 2021 Apr 8;11(4):e045922. doi: 10.1136/bmjopen-2020-045922 (PMC8039279; doi:10.1136/bmjopen-2020-045922)
Supplement: Author's manuscript [file bmjopen-2020-045922.draft_revisions.pdf]

BMJ Open is committed to open peer review. As part of this commitment we make the peer review history of every article we publish publicly available.

When an article is published we post the peer reviewers' comments and the authors' responses online. We also post the versions of the paper that were used during peer review. These are the versions that the peer review comments apply to.

The versions of the paper that follow are the versions that were submitted during the peer review process. They are not the versions of record or the final published versions. They should not be cited or distributed as the published version of this manuscript.

BMJ Open is an open access journal and the full, final, typeset and author-corrected version of record of the manuscript is available on our site with no access controls, subscription charges or pay-per-view fees (http://bmjopen.bmj.com).

If you have any questions on BMJ Open's open peer review process please email info.bmjopen@bmj.com

BMJ Open

Acceptability and Feasibility of a Nutrigenetics-based Intervention Approach for Adults with Non-Alcoholic Fatty Liver Disease (NAFLD): Study Protocol for a Randomised Controlled Feasibility Trial

Journal:	*BMJ Open*
Manuscript ID	bmjopen-2020-045922
Article Type:	Protocol
Date Submitted by the Author:	21-Oct-2020

Complete List of Authors:	Haigh, Laura; Newcastle Upon Tyne Hospitals NHS Trust, Regional Liver Unit; Newcastle University, Faculty of Medical Sciences<br>McPherson, Stuart; Newcastle Upon Tyne Hospitals NHS Trust, Regional Liver Unit; Newcastle University, Faculty of Medical Sciences<br>Mathers, John; Newcastle University, Human Nutrition Research Centre<br>Anstee, Quentin; Newcastle University, Faculty of Medical Sciences; Newcastle Upon Tyne Hospitals NHS Trust, Regional Liver Unit

Keywords:	Hepatology < INTERNAL MEDICINE, NUTRITION & DIETETICS, THERAPEUTICS

SCHOLARONE™
Manuscripts

I, the Submitting Author has the right to grant and does grant on behalf of all authors of the Work (as defined in the below author licence), an exclusive licence and/or a non-exclusive licence for contributions from authors who are: i) UK Crown employees; ii) where BMJ has agreed a CC-BY licence shall apply, and/or iii) in accordance with the terms applicable for US Federal Government officers or employees acting as part of their official duties; on a worldwide, perpetual, irrevocable, royalty-free basis to BMJ Publishing Group Ltd ("BMJ") its licensees and where the relevant Journal is co-owned by BMJ to the co-owners of the Journal, to publish the Work in this journal and any other BMJ products and to exploit all rights, as set out in our licence.

The Submitting Author accepts and understands that any supply made under these terms is made by BMJ to the Submitting Author unless you are acting as an employee on behalf of your employer or a postgraduate student of an affiliated institution which is paying any applicable article publishing charge ("APC") for Open Access articles. Where the Submitting Author wishes to make the Work available on an Open Access basis (and intends to pay the relevant APC), the terms of reuse of such Open Access shall be governed by a Creative Commons licence – details of these licences and which Creative Commons licence will apply to this Work are set out in our licence referred to above.

Other than as permitted in any relevant BMJ Author's Self Archiving Policies, I confirm this Work has not been accepted for publication elsewhere, is not being considered for publication elsewhere and does not duplicate material already published. I confirm all authors consent to publication of this Work and authorise the granting of this licence.

For peer review only

Acceptability and Feasibility of a Nutrigenetics-based Intervention Approach for Adults with Non-Alcoholic Fatty Liver Disease (NAFLD): Study Protocol for a Randomised Controlled Feasibility Trial

[1,2,3]Laura Haigh, [1,2] Stuart McPherson, [3] John C Mathers*, [1,2] Quentin M Anstee*
*Joint Senior Authors

Author affiliations
[1]Regional Liver Unit, Newcastle upon Tyne Hospitals NHS Foundation Trust, Newcastle upon Tyne, UK.
[2] Translational and Clinical Research Institute, Faculty of Medical Sciences, Newcastle University, Newcastle upon Tyne, UK.
[3] Human Nutrition Research Centre, Population Health Sciences Institute, Faculty of Medical Sciences, Newcastle University, Newcastle upon Tyne, UK.

Correspondence: Laura Haigh, Regional Liver Unit, Newcastle upon Tyne Hospitals NHS Foundation Trust, Newcastle upon Tyne, UK. NE7 7DN. Email: l.haigh3@newcastle.ac.uk Tel: 0191 2231568 Fax: 0191 208 0723

Keywords: Non-alcoholic Fatty Liver Disease; Nutrigenetics; Metabolomics; Mediterranean diet
Word count: 3347

For peer review only

Abstract

Introduction: Lifestyle interventions targeting weight loss and improved dietary patterns are the recommended treatment for non-alcoholic fatty liver disease (NAFLD). However, the effectiveness of current established diet therapies is sub-optimal. The *PNPLA3* gene modifies disease outcome and hepatic lipid handling, but the role of *PNPLA3* variants in modulating responsiveness to different diet therapies is unknown.

Methods and analysis: This project aims to assess the feasibility of conducting a genotype-driven randomised controlled trial (RCT) investigating the differential response to a Mediterranean Diet (MD) intervention of NAFLD patients according to genotype for the rs738409 (I148M) variant of *PNPLA3*. A single centre randomised controlled feasibility trial will be undertaken. We will recruit 60 adults with NAFLD from a tertiary hepatology centre in England. In a cross-over design, participants will undertake Diet 1 (MD) and Diet 2 (control) for 4-weeks, in random order (1:1 allocation), separated by a 4-weeks washout period. Participants will complete one-to-one diet and lifestyle consultations at baseline, end of diet phase 1, end of washout and end of diet phase 2. Participants will be advised to maintain baseline levels of physical activity and body weight. The primary outcome is the acceptability and feasibility of the intervention protocol. Secondary outcomes include exploratory assessment of liver fibrosis biomarkers and lipid biomarkers.

Ethics and dissemination: Ethical approval was granted by East of Scotland Research Ethics Service REC 1 (19/ES/0112). Results will be disseminated through peer-reviewed journals and presented at local, national and international meetings and conferences. The findings of this trial will lay the foundation for a future definitive RCT by informing trial design and optimising the intervention diets, instruments and procedures.

Clinical trial registration: ISRCTN93410321

For peer review only

For peer review only - http://bmjopen.bmj.com/site/about/guidelines.xhtml

Article Summary

Strengths and limitations of this study

- The main strength of this trial is the use of explicit feasibility and acceptability criteria as primary outcomes.
- A unique feature of this trial is the use of metabolomics biomarkers to: i) capture diet-mediated effects on lipid metabolism and ii) provide objective assessment of dietary intake.
- In the definitive RCT that is expected to follow this feasibility trial, the use of a cross-over design will facilitate more precise comparisons between intervention/control diets on a within-participant basis.
- The effectiveness of washout to return outcome variables to baseline will be evaluated.
- The MD intervention has been systematically developed and informed by research evidence and patient and public feedback.
- This trial will generate qualitative and quantitative information to establish the feasibility and acceptability of the protocol.

For peer review only

For peer review only - http://bmjopen.bmj.com/site/about/guidelines.xhtml

Introduction

Non-alcoholic fatty liver disease (NAFLD) affects 25% of the global population (1, 2) and is a spectrum spanning steatosis (non-alcoholic fatty liver, NAFL), through steatosis plus inflammation, hepatocyte damage and progressive fibrosis (non-alcoholic steatohepatitis, NASH), that may potentially develop into cirrhosis and hepatocellular carcinoma (3-5). NAFLD is strongly associated with obesity and cardio-metabolic disease (2, 6).

NAFLD attributed rates of advanced liver disease and transplantation are increasing, but effective pharmacotherapy is unavailable (2, 3). A major challenge is the substantial inter-individual variation in NAFLD susceptibility, progression and outcome, due partly to gene-environment interactions (2, 3). The patatin-like phospholipase domain containing 3 *(PNPLA3)* rs738409 single nucleotide polymorphism is a common modifier of disease outcome and its impact is amplified by adiposity (7-10). This additive effect poses considerable concern, as patients with NAFLD tend to consume excess calories as a consequence of poor-quality diets and sedentary lifestyles (11-13).

The PNPLA3 protein has lipase activity and regulates lipid droplets in hepatocytes and hepatic stellate cells (14). Several studies have contributed to understanding of its function in hepatic lipid handling (15). Numerous genome-wide association studies (GWAS) have shown its association with the entire disease spectrum, which has been confirmed in various populations (8, 10, 12, 16, 17). Therefore, among the identified NAFLD-related genes, *PNPLA3* has the most potential as a therapeutic target for NAFLD (8, 10, 12).

The main treatment recommendations for NAFLD are lifestyle interventions targeting weight loss and improved dietary patterns (4, 5). The Mediterranean diet (MD) reduces steatosis, improves liver biochemistry and cardio-metabolic dysfunction, with or without weight loss (18-23). However, there is sub-optimal response to current diet therapies and more effective approaches for enhancing adherence to diet therapies is needed (11).

Personalised nutrition approaches that use information on individual participant characteristics to tailor the dietary intervention may improve the sub-optimal response to current diet therapies (24). Differences in gene sequence can alter the activity of encoded proteins and affect the response of individuals to dietary components (25). Emergent research has shown nutritional regulation of *PNPLA3* (15, 25) so that patients with NAFLD carrying the *PNPLA3* risk allele might benefit more from weight loss but less from omega-3 supplementation (25-28). However, the role of *PNPLA3* variants in influencing responsiveness to different diet therapies is poorly understood.

This project aims to determine whether it is feasible to conduct a RCT to investigate the impact of *PNPLA3* carriage on responsiveness to MD and NAFLD severity and to provide preliminary data to inform the development of a definitive RCT.

For peer review only - http://bmjopen.bmj.com/site/about/guidelines.xhtml

Methods and analysis

Study design and setting

This trial is a single centre, randomised controlled feasibility trial. In a cross-over design, participants will be randomised to either Diet 1 (MD) or Diet 2 (control) for 4-weeks, in random order, separated by a 4-weeks washout period. All study visits will be conducted in the outpatient hepatology services, in The Newcastle upon Tyne Hospitals NHS Foundation Trust, (NuTH) United Kingdom. An overview of the study design is provided in Figure 1.

Study objectives

The primary objective is to determine whether the protocol for a future definitive RCT is acceptable and feasible. We will:

- Determine the feasibility of recruitment and retention.
- Determine the acceptability of the diets, instruments and procedures.
- Evaluate adherence to, and completion of, the diets and procedures.
- Evaluate implementation fidelity and how practicable it is to deliver the protocol in a clinical setting.
- Estimate outcome variability.

Secondary objectives include collection of preliminary exploratory data on liver fibrosis biomarkers and lipid biomarkers, to determine if *PNPLA3* carriage influences response to diet treatments.

Sampling and recruitment

A recruitment target of 60 individuals with either imaging or histological evidence of NAFLD was established in accordance with published guidance (29). This sample size is estimated to provide information to assess critical feasibility and acceptability indicators with the necessary degree of precision (29). Adults with NAFLD will be recruited from a tertiary hepatology centre in NuTH, which covers a population of approximately 3million in Northern United Kingdom. Potential participants meeting the inclusion and exclusion criteria will be identified from NuTH hepatology clinic lists and electronic records by members of the research and clinical care team. Potential participants will be approached during an appointment in liver outpatients or through screening of clinical notes and invited to participate in the study. The research team will contact patients after 48 hours to answer any queries. Enrolment will follow the receipt of full and written informed consent and successful screening. Recruitment and intervention delivery are anticipated to take place over 12 months.

Inclusion and exclusion criteria

Participant eligibility criteria are detailed in Table 1. We have chosen minimal exclusions to reflect the target population for a subsequent definitive RCT.

Table 1. Patient eligibility criteria

Inclusion criteria

For peer review only - http://bmjopen.bmj.com/site/about/guidelines.xhtml

• 18-80 years old with NAFLD (confirmed on liver biopsy or by clinical diagnosis with imaging evidence of steatosis).
• Weekly alcohol consumption <14(women)/ <21(men) units in the last 24months.
• Weight stable (+/-5%) for previous 3 months.
• Capacity to provide informed consent.
• Ability to write and converse in English without assistance of an interpreter.
Exclusion criteria
• All cancers within 5 years (except squamous cell carcinoma).
• Evidence of co-existent liver disease/ presence of secondary causes of NAFLD (except Gilbert's syndrome).
• Decompensated NASH-cirrhosis (Child Pugh >6).
• Uncontrolled psychiatric disorder (e.g. acute psychosis).
• Uncontrolled medical condition (e.g. HbA1c ≥80mmol/l or acute coronary event or stroke within 12 months).
• Active eating disorder.
• Active substance misuse.
• Other prescribed dietary regimens, food intolerances and/or food allergies.
• Mediterranean diet score (MEDAS) >8 (high MD consumption).
• Previous weight loss surgery.
• Taking anti-obesity medications and/ or engaged in structured, multi-component weight management interventions (specialist, community or commercial providers).
• Insulin use.
• Pregnancy/ lactation.

Randomisation and allocation strategy: Eligible participants will be allocated in a 1:1 ratio to receive either Diet 1 or Diet 2 first using computer generated randomisation. Pre-randomisation genotyping for specific genetic variants (*PNPLA3 rs738409*) will be conducted using blood samples collected at screening. Interim analysis of the genotype distribution amongst recruited cohort will be conducted at intervals as recruitment proceeds (e.g. after one third and two-thirds have been randomised); randomisation will then be stratified by *PNPLA3* status at baseline to ensure a balanced recruitment for *PNPLA3 rs738409* genetic status (wildtype, heterozygote, homozygote).

The major allele frequency for *PNPLA3 rs738409* is approximately 0.25 in the UK general population (30) and the minor allele frequency (MAF) is greater in those with NAFLD (17). Accordingly, we expect to reach the recruitment target for wild-type cases sooner than for carriers of the genetic variant and so some patients with a specific genotype may not randomised if sufficient cases of a given genotype are already enrolled. Those discharged from the study will be offered a one-to-one nutrition education and counselling

For peer review only

For peer review only - http://bmjopen.bmj.com/site/about/guidelines.xhtml

consultation with a dietitian. It will not be possible to blind participants, clinicians or research investigators to which diet each participant is on in each study period.

Trial procedures

The trial procedures are outlined in Table 2. At an initial screening appointment, demographic, clinical, dietary and lifestyle data will be collected to determine patient eligibility and a blood sample will be taken for *PNPLA3 rs738409* genotyping. In addition, at baseline, liver steatosis will be assessed using Controlled Attenuation Parameter (CAP) measured contemporaneously with liver stiffness by Transient Elastography, Fibroscan®.

Enrolled participants will complete one-to-one diet and lifestyle consultations at baseline, end of diet phase 1 (4-weeks), end of washout (8-weeks) and end of diet phase 2 (12-weeks). At each of these time-points, the following measures will be taken; anthropometry and body composition; blood biochemistry; dietary intake, urine samples and physical activity. The clinical status and medication consumption of participants will be checked at each time-point. Patient-reported outcome data will be captured at the end of each diet phase (4-weeks and 12-weeks).

Dietary intervention and control treatments

The experimental Diet 1 is a Mediterranean-style diet (MD) based on the traditional MD and MD pyramid (31). The MD is characterised by a high quantity of plant-based foods, unrefined cereals, fruit and vegetables, olive oil, and nuts; eating white meat, fish and legumes in moderation; restricting red and processed meats, and sweets; and drinking red wine moderately (32). The diet was designed to be easy to follow over 4 weeks, informed by research evidence (33, 34) and the findings from our earlier pilot study with patients and the public that explored barriers and facilitators to adoption of a MD intervention (34). They highlighted the importance of reducing the burden of dietary changes, diet supporters in the household, regular nutritional counselling and preference for face-to-face contacts. These ideas have been incorporated into the final design.

To reduce participant burden and facilitate changes in food environment, some intervention foods will be supplied as pre-packaged ready meals (FreshPrepare©) and extra virgin olive oil 750ml (Filippo Berio©). Ten pre-packaged ready meals will be home delivered weekly, taken as two main meals per day (lunch and evening meal) for five days. The meals can be ordered online, and examples of the available options are detailed in Appendix Table 1. The provision of these intervention foods will assist in standardising food consumption and minimise variability in dietary intake.

To enhance MD adoption, nutrition counselling and education will be provided one-to-one to participants during visits, by a dietitian. This will include advice on the selection and preparation of appropriate meals, snacks and drinks. The consultation incorporates the ‘model and process for nutrition and dietetic practice’,

For peer review only - http://bmjopen.bmj.com/site/about/guidelines.xhtml

(35) and involves participant discussion. Personalisation of the diet for specific personal and sociocultural preferences will be provided.

A patient information booklet has been designed to explain NAFLD, the principles of MD, and how it can be successfully followed. This will be given to participants as evidence-based written material from a credible source. Behaviour change techniques will be utilised to increase intervention effectiveness. These include barrier identification, problem solving, goal setting, and action planning; social support; and instruction on how to perform a behaviour and behavioural practice (36-38).

Diet 2 (control) will involve counselling participants to consume their habitual diet. During washout participants will be asked to return to their habitual diet. Participants will be asked to maintain baseline levels of physical activity and body weight (+/-3%) throughout the trial duration. The dietitian will contact participants by telephone mid-way through each diet phase to review progress, provide additional counselling and answer any queries.

Table 2. Schedule of study measures

Variable	Instrument	Timepoint				
		Screening	Baseline	End of diet phase 1	End of washout	End of diet phase 2
Inclusion/ exclusion criteria		×				
Informed consent		×				
Demographic data and medical history	Self-report and clinical records	×				
Genotyping	Venous blood sample	×				
Anthropometrics: weight, height, waist and hip circumference			×	×	×	×
Whole body composition	Bioelectrical Impedance Analysis		×	×	×	×
Cardio-metabolic measures: glucose, insulin, HbA1c, lipid profile and blood pressure	Fasting venous blood samples		×	×	×	×
Liver function: LFTs, Ferritin, FBC, CRP, lipid biomarkers	Fasting venous blood samples		×	×	×	×
Liver steatosis: CAP	TE Fibroscan™		×			
Liver fibrosis:	TE Fibroscan™		×			

For peer review only - http://bmjopen.bmj.com/site/about/guidelines.xhtml

Liver stiffness, and PRO-C3	Fasting venous blood samples		×	×	×	×
Patient-reported outcomes	EQ5D, CLDQ-NASH and NASH-CHECK			×		×
Physical activity	Accelerometer		×	×	×	×
Dietary intake: dietary biomarkers, diet recall and MD questionnaire	Urine samples		×	×	×	×
	INTAKE24		×	×	×	×
	MEDAS		×	×	×	×

Abbreviations: HbA1c, glycated haemoglobin; LFTs, liver function tests; FBC, full blood count; CRP, C-Reactive Protein; CAP, controlled attenuation parameter; TE, transient elastography; PRO-C3, N-protease cleaved PIIINP neo-epitope; EQ-5D, euroqol five dimension scale; CLDQ-NASH, chronic liver disease questionnaire for non-alcoholic steatohepatitis; MEDAS, Mediterranean diet assessment score.

For peer review only

Outcome assessment and process evaluation: This mixed methods feasibility trial will generate qualitative and quantitative information about feasibility and acceptability to inform a future definitive RCT. The following criteria will be assessed:

- The feasibility of recruitment and retention

The consent rate (the number of eligible participants who consent divided by the total number who are eligible and who are invited to participate), the recruitment rate (the number of participants recruited per month), and the retention rate (the number of participants who complete follow-up data collection divided by the total number randomised) will be assessed using a trial log between baseline and 12 months.

- The acceptability of the diets, instruments and procedures

Patient-reported outcome data will be used to identify impact of diet treatments and trial procedures on quality of life using the PROs 'EQ5D', 'CLDQ-NASH' and 'NASH-CHECK' (39-41). At trial end, open-response questions will be used to capture perceptions of: i) the randomisation procedure, ii) the, acceptability of the MD intervention and iii) the components of the measurement protocol. These data will be recorded, and thematic analyses performed.

- Adherence to, and completion of, the diets and procedures

Participant adherence to trial procedures will be assessed by tracking the number of completed visits, and the completeness of data collection will be assessed using the trial log between baseline and 12 weeks. Diet adherence will be assessed in self-report measures; Mediterranean diet assessment score (MEDAS) is based on a small number of foods measured in servings/d or servings/week. Scores range between 0-14, and can be categorised as low, moderate or high consumption (<5, 6-9 and >10 points, respectively) (42). Dietary intake will be quantified using INTAKE24, an open-source computerised dietary recall system based on multiple-pass 24-hour recall (43, 44). INTAKE24 data will be converted to MD scores and Dietary Inflammatory Index™ will be calculated (45).

For peer review only - http://bmjopen.bmj.com/site/about/guidelines.xhtml

In addition, dietary biomarkers will be quantified in urine to provide objective measures of dietary intake, without self-report bias (46). Three spot urine samples will be collected at baseline and after each diet treatment on non-consecutive days, including one weekend day, to provide estimates of habitual dietary intake. The three urine samples from each time-point will be pooled and analysed using Ultra High-Performance Liquid Chromatography (UHPLC) (47).

- Implementation fidelity and how practicable protocol processes are to deliver in a clinical setting

Data collection burden will be measured as the time taken to administer protocol processes. Participant processing time is the number of days from initial contact to enrolment. Both will be assessed using the trial log between baseline and 12 months. To assess integrity and fidelity, a trial protocol checklist will be monitored with missing, incomplete or unreliable data recorded between baseline and 12 months.

- Preliminary exploratory data on liver fibrosis biomarkers and lipid biomarkers

In collaboration with partners in the EU IMI2-funded LITMUS biomarkers consortium (https://litmus-project.eu/), plasma will be analysed to characterise changes in the lipidome and metabolome to help elucidate mechanisms underlying specific changes in lipid metabolism. Plasma PRO-C3 concentration, as a marker of fibrogenesis, will be measured using competitive ELISAs (48).

The data from this feasibility trial will be reported as descriptive summaries. Sample characteristics including age, gender, ethnicity, medical history and disease sub-classification and severity will be presented. Although the trial is not powered to detect significant changes in clinical and lifestyle outcomes, key variables of interest (outlined in schedule of assessments) will be monitored and any preliminary changes reported. These data will determine the feasibility of testing components and enable the statistical power calculations for a subsequent RCT. The balance of the groups after randomisation will be explored. Descriptive statistics ((mean, standard deviation (SD) and counts (percentage)) will summarise continuous and categorical data as appropriate. No imputation of missing data will be undertaken.

Success criteria

The feasibility indicators are binary (successful or unsuccessful). “Successful” would indicate the protocol is sufficiently robust to advance to a fully powered definitive RCT, whilst “unsuccessful” indicates that protocol changes are required (49).

- The acceptability of diets, instruments and procedures.
- Consent rate (25% of individuals consenting).
- Recruitment rate (target 60 individuals/9months) (acceptable 45 individuals/9months).
- Retention rate (target 45 individuals/3months) (acceptable 36 individuals/3months).
- Participant adherence (75% completed visits/data collection).
- Data collection burden (target >75% of individuals≤1.5h) (acceptable >75% of individuals<2hr).

For peer review only - http://bmjopen.bmj.com/site/about/guidelines.xhtml

- Participant processing time (mean time <14days between initial contact to enrolment).
- Trial protocol administration (<10% deviation from checklist).

Patient and public involvement

The early stages of the research process involving the preparation of the research proposal were supported by a national liver patient support group (LIVErNORTH). LIVErNORTH collaborate on the joint production of the patient information booklet, which is used in one-to-one diet and lifestyle consultations. To enhance the development of the MD intervention the findings from an earlier pilot study (34) and research evidence were discussed with a patient panel (APEX) as well as patients attending clinical services in NuTH. APEX advised on the patient experience and assessed the burden of the trial instruments and procedures. This patient and public feedback was integrated into the final design. There are plans in the future to involve patients and the public as the trial progresses, guiding the dissemination of trial results to participants and relevant wider patient communities.

Discussion

There is an urgent need to identify potential therapeutic interventions that can prevent NAFLD progression and induce regression (11). Lifestyle interventions to induce weight loss and improve dietary lifestyle patterns are the mainstay of NAFLD treatment (4, 5). However, diet lifestyle targets are often difficult to achieve in practice and definitive data are needed on the optimal strategies to enhance adherence to diet treatments (11). Stratified and targeted diet therapies may be an advance on the relatively ineffective 'one-size fits all' treatments. To that end, there is a need to explore the underlying mechanisms through which genotype influences responses to dietary components, and the subsequent effects on NAFLD.

The primary objective of this trial is to determine the acceptability and feasibility of a nutrigenetic approach for adults with NAFLD. This trial adopts a mixed methods approach designed to establish whether the protocol is sufficiently robust and to inform any necessary refinements to diets, instruments and procedures. In addition to the primary outcomes, the trial will provide data on variability of secondary outcomes for power calculations to inform the design of a subsequent RCT.

A unique feature of this trial is the use of metabolomics biomarkers to: i) capture diet-mediated effects on lipid metabolism and ii) provide objective assessment of dietary intake. Metabolomics approaches provide novel insights into complex disease traits and provide a powerful tool to predict and monitor responsiveness to diet treatments for NAFLD (50). The application of 'omics' could support the design of innovative and effective diet therapies across the full NAFLD spectrum, and to understand their mechanisms (50). The use of self-reported dietary intake assessment methods presents significant challenges both in research and clinical practice (51) and some of these challenges can be overcome using urine-based biomarker approaches (46). Thus, we will combine self-reported data with the quantification of urinary dietary biomarkers to overcome this weakness.

For peer review only

For peer review only - http://bmjopen.bmj.com/site/about/guidelines.xhtml

The main strength of this trial is the use of explicit feasibility and acceptability criteria as primary outcomes. Patient-reported outcome measures and open-response questions will be especially important to identify impact of diet treatments and trial procedures on quality of life and individuals' perceptions of trial participation. In addition, the use of a cross-over design will facilitate more precise comparisons between intervention/control diets on a within-participant basis (52). This design is favoured in short-term trials of long-term conditions with intermediate outcomes (52). Nevertheless, a limitation of this design is the possibility of carry-over effects from one experimental period to the next. We have addressed this issue by including a 4-week washout period. The required duration of washout period is influenced by the nature and duration of the intervention but, in nutritional cross-over studies, 2-4 weeks is often sufficient (53, 54). This is confirmed by studies of metabolomics biomarkers in urine which show that these respond rapidly to dietary change (55). Importantly, we will evaluate the effectiveness of washout to return outcome variables to baseline. Additionally, participants will be supplied with intervention foods and counselled to return to habitual dietary intake during washout. Advice will be given to maintain baseline levels of physical activity and body weight (+/-3%) throughout. The intervention period (4-weeks) is relatively short and is unlikely to reveal the full effects of the dietary intervention on markers of liver health. However, this study is designed, primarily, to provide information on the acceptability and feasibility of the study protocol. Finally, the MD intervention in this trial has been systematically developed and informed by research evidence and patient and public feedback.

This trial will assess the feasibility of conducting a genotype-driven RCT investigating the differential response to a MD intervention of patients according to genotype for the rs738409 (I148M) variant of *PNPLA3*. The findings of this trial will lay the foundation for a future definitive RCT by informing trial design and optimising the intervention diets, instruments and procedures. In the longer run, the outcomes of this research programme may lead to fewer NAFLD patients who need intensified medical treatment, reducing cost burden, and premature mortality and morbidity.

Ethics and dissemination

Ethical approval was granted by East of Scotland Research Ethics Service REC 1 (19/ES/0112). NHS Research and Development approval was granted by the Newcastle upon Tyne Hospitals NHS Foundation Trust (R&D8985). This trial will be conducted to a high standard in accordance with the protocol, the principles of good clinical practice (GCP), relevant regulations, guidelines and with regard to patient safety and welfare. The findings of this trial will be submitted for publication in peer-reviewed journals and presented at local, national and international meetings and conferences. A lay summary of results will be available for all study participants.

Trial status

The first participant was enrolled on the 11th February 2020 with recruitment expected to be completed by the 31st March 2021. The recruitment period has been extended to mitigate the potential impact of COVID-19 on the research plans.

For peer review only - http://bmjopen.bmj.com/site/about/guidelines.xhtml

References

1. Younossi ZM, Koenig AB, Abdelatif D, Fazel Y, Henry L, Wymer M. Global epidemiology of nonalcoholic fatty liver disease-Meta-analytic assessment of prevalence, incidence, and outcomes. Hepatology. 2016;64(1):73-84.
2. Younossi Z, Tacke F, Arrese M, Chander Sharma B, Mostafa I, Bugianesi E, et al. Global Perspectives on Nonalcoholic Fatty Liver Disease and Nonalcoholic Steatohepatitis. Hepatology. 2019;69(6):2672-82.
3. Anstee QM, Targher G, Day CP. Progression of NAFLD to diabetes mellitus, cardiovascular disease or cirrhosis. Nat Rev Gastroenterol Hepatol. 2013;10(6):330-44.
4. Chalasani N, Younossi Z, Lavine J, Mae Diehl A, Brunt E, Cusi K, et al. The diagnosis and management of non-alcoholic fatty liver disease: Practice guideline by the American Gastroenterological Association, American Association for the Study of Liver Diseases, and American College of Gastroenterology. Gastroenterology. 2012;143(2):503.
5. European Association for the Study of the Liver (EASL), European Association for the Study of Diabetes (EASD), European Association for the Study of Obesity (EASO). EASL–EASD–EASO Clinical Practice Guidelines for the management of non-alcoholic fatty liver disease. Hepatology. 2016;64:1388–402.
6. Younossi Z, Anstee QM, Marietti M, Hardy T, Henry L, Eslam M, et al. Global burden of NAFLD and NASH: trends, predictions, risk factors and prevention. Nat Rev Gastroenterol Hepatol. 2016;15(1):11-20.
7. Anstee QM, Day CP. The Genetics of Nonalcoholic Fatty Liver Disease: Spotlight on PNPLA3 and TM6SF2. Semin Liver Dis. 2015;35(3):270-90.
8. Liu YL, Patman GL, Leathart JB, Piguet AC, Burt AD, Dufour JF, et al. Carriage of the PNPLA3 rs738409 C >G polymorphism confers an increased risk of non-alcoholic fatty liver disease associated hepatocellular carcinoma. J Hepatol. 2014;61(1):75-81.
9. Stender S, Kozlitina J, Nordestgaard BG, Tybjaerg-Hansen A, Hobbs HH, Cohen JC. Adiposity amplifies the genetic risk of fatty liver disease conferred by multiple loci. Nat Genet. 2017;49(6):842-7.
10. Mann JP, Anstee QM. NAFLD: PNPLA3 and obesity: a synergistic relationship in NAFLD. Nat Rev Gastroenterol Hepatol. 2017;14(9):506-7.
11. Romero-Gomez M, Zelber-Sagi S, Trenell M. Treatment of NAFLD with diet, physical activity and exercise. Journal of Hepatology. 2017;67(4):829-46.
12. Dong XC. PNPLA3-A Potential Therapeutic Target for Personalized Treatment of Chronic Liver Disease. Frontiers in medicine. 2019;6:304-.
13. Gerber L, Otgonsuren M, Mishra A, Escheik C, Birerdinc A, Stepanova M, et al. Non-alcoholic fatty liver disease (NAFLD) is associated with low level of physical activity: a population-based study. Aliment Pharmacol Ther. 2012;36:772–81.
14. Pingitore P, Romeo S. The role of PNPLA3 in health and disease. Biochimica et Biophysica Acta (BBA) - Molecular and Cell Biology of Lipids. 2019;1864(6):900-6.
15. Basu Ray S. PNPLA3-I148M: a problem of plenty in non-alcoholic fatty liver disease. Adipocyte. 2019;8(1):201-8.
16. Valenti L, Al-Serri A, Daly AK, Galmozzi E, Rametta R, Dongiovanni P, et al. Homozygosity for the patatin-like phospholipase-3/adiponutrin I148M polymorphism influences liver fibrosis in patients with nonalcoholic fatty liver disease. Hepatology. 2010;51(4):1209-17.
17. Anstee QM, Darlay R, Cockell S, Meroni M, Govaere O, Tiniakos D, et al. Genome-wide association study of non-alcoholic fatty liver and steatohepatitis in a histologically-characterised cohort. Journal of Hepatology. 2020.
18. Ryan MC, Itsiopoulos C, Thodis T, Ward G, Trost N, Hofferberth S, et al. The Mediterranean diet improves hepatic steatosis and insulin sensitivity in individuals with non-alcoholic fatty liver disease. Journal of hepatology. 2013;59(1):138-43.
19. Katsagoni CN, Egkomiti A, Papageorgiou M, Ioannidou P, Fragopoulou E, Papatheodoridis G, et al. Improvement in liver function after an intervention based on the Mediterranean diet in patients with non alcoholic fatty liver disease (NAFLD). Clinical Nutrition ESPEN. 2016;13:e57.
20. Gelli C, Tarocchi M, Abenavoli L, Di Renzo L, Galli A, De Lorenzo A. Effect of a counseling-supported treatment with the Mediterranean diet and physical activity on the severity of the non-alcoholic fatty liver disease. World J Gastroenterol. 2017;23(17):3150-62.
21. Aller R, Izaola O, de la Fuente B, de Luis D. Mediterranean diet is associated with liver histology in patients with non alcoholic fatty liver disease. Nutricion Hospitalaria. 2015;32(6):2518-24.
22. Shai I, Schwarzfuchs D, Henkin Y, Shahar DR, Witkow S, Greenberg I, et al. Weight Loss with a Low-Carbohydrate, Mediterranean, or Low-Fat Diet. New England Journal of Medicine. 2008;359(3):229-41.

For peer review only - http://bmjopen.bmj.com/site/about/guidelines.xhtml

23. Properzi C, O'Sullivan TA, Sherriff JL, Ching HL, Jeffrey GP, Buckley RF, et al. Ad Libitum Mediterranean and Low-Fat Diets Both Significantly Reduce Hepatic Steatosis: A Randomized Controlled Trial. Hepatology. 2018;68(5):1741-54.
24. Ordovas JM, Ferguson LR, Tai ES, Mathers JC. Personalised nutrition and health. Bmj. 2018;361:bmj.k2173.
25. Dongiovanni P, Valenti L. A Nutrigenomic Approach to Non-Alcoholic Fatty Liver Disease. Int J Mol Sci. 2017;18(7):1534.
26. Scorletti E, West AL, Bhatia L, Hoile SP, McCormick KG, Burdge GC, et al. Treating liver fat and serum triglyceride levels in NAFLD, effects of PNPLA3 and TM6SF2 genotypes: Results from the WELCOME trial. J Hepatol. 2015;63(6):1476-83.
27. Kalafati I, al. e. Genetics of Nonalcoholic Fatty Liver Disease: Role of Diet as a modifying factor Clin Nut. 2014;3:223-32.
28. Shen J, Wong GL, Chan HL, Chan RS, Chan HY, Chu WC, et al. PNPLA3 gene polymorphism and response to lifestyle modification in patients with nonalcoholic fatty liver disease. J Gastroenterol Hepatol. 2015;30(1):139-46.
29. Lancaster GA, Dodd S, Williamson PR. Design and analysis of pilot studies: recommendations for good practice. Journal of Evaluation in Clinical Practice. 2004;10(2):307-12.
30. Ensembl. Variant: rs738409 SNP, 2020, April, [Available from: https://apr2020.archive.ensembl.org/Homo_sapiens/Variation/Population?db=core;r=22:43928347-43929347;v=rs738409;vdb=variation;vf=88266706.
31. Bach-Faig A, Berry EM, Lairon D, Reguant J, Trichopoulou A, Dernini S, et al. Mediterranean diet pyramid today. Science and cultural updates. Public Health Nutrition. 2011;14(12A):2274-84.
32. Anania C, Perla FM, Olivero F, Pacifico L, Chiesa C. Mediterranean diet and nonalcoholic fatty liver disease. World J Gastroenterol. 2018;24(19):2083-94.
33. Wareham N. Fenland Study investigates interaction between genetic and lifestyle factors in determining obesity, diabetes and related metabolic disorders which present a considerable public health concern. . ISRCTN register 2013.
34. Haigh L, Bremner S, Houghton D, Henderson E, Avery L, Hardy T, et al. Barriers and Facilitators to Mediterranean Diet Adoption by Patients With Nonalcoholic Fatty Liver Disease in Northern Europe. Clin Gastroenterol Hepatol. 2019;17(7):1364-71.e3.
35. The British Dietetic Association. Model and Process for Nutrition and Dietetic Practice,. 2016, May.
36. Lara J, Evans EH, O'Brien N, Moynihan PJ, Meyer TD, Adamson AJ, et al. Association of behaviour change techniques with effectiveness of dietary interventions among adults of retirement age: a systematic review and meta-analysis of randomised controlled trials. BMC medicine. 2014;12:177-.
37. Cradock KA, ÓLaighin G, Finucane FM, Gainforth HL, Quinlan LR, Ginis KAM. Behaviour change techniques targeting both diet and physical activity in type 2 diabetes: A systematic review and meta-analysis. International Journal of Behavioral Nutrition and Physical Activity. 2017;14(1):18.
38. Michie S, Richardson M, Johnston M, Abraham C, Francis J, Hardeman W, et al. The behavior change technique taxonomy (v1) of 93 hierarchically clustered techniques: building an international consensus for the reporting of behavior change interventions. Ann Behav Med. 2013;46(1):81-95.
39. Devlin NJ, Brooks R. EQ-5D and the EuroQol Group: Past, Present and Future. Applied Health Economics and Health Policy. 2017;15(2):127-37.
40. Younossi ZM, Stepanova M, Younossi I, Racila A. Validation of Chronic Liver Disease Questionnaire for Nonalcoholic Steatohepatitis in Patients With Biopsy-Proven Nonalcoholic Steatohepatitis. Clin Gastroenterol Hepatol. 2019.
41. Doward L, Balp MM, Twiss J, Slota C, Cryer D, Langford A, et al. Measuring what matters to patients: the development of the NASH-CHECK, a new patient-reported outcome instrument for nonalcoholic steatohepatitis. Journal of Hepatology. 2018;68:S570-S.
42. Martínez-González MA, García-Arellano A, Toledo E, Salas-Salvadó J, Buil-Cosiales P, Corella D, et al. A 14-item Mediterranean diet assessment tool and obesity indexes among high-risk subjects: the PREDIMED trial. PloS one. 2012;7(8):e43134-e.
43. Simpson E, Bradley J, Poliakov I, Jackson D, Olivier P, Adamson AJ, et al. Iterative Development of an Online Dietary Recall Tool: INTAKE24. Nutrients. 2017;9(2).
44. Foster E, Lee C, Imamura F, Hollidge SE, Westgate KL, Venables MC, et al. Validity and reliability of an online self-report 24-h dietary recall method (Intake24): a doubly labelled water study and repeated-measures analysis. J Nutr Sci. 2019;8:e29.

For peer review only - http://bmjopen.bmj.com/site/about/guidelines.xhtml

45. Shivappa N, Steck SE, Hurley TG, Hussey JR, Hébert JR. Designing and developing a literature-derived, population-based dietary inflammatory index. Public health nutrition. 2014;17(8):1689-96.
46. Lloyd AJ, Wilson T, Willis ND, Lyons L, Phillips H, Janssen HG, et al. Developing community-based urine sampling methods to deploy biomarker technology for the assessment of dietary exposure. Public Health Nutr. 2020:1-12.
47. Lloyd AJ, Willis ND, Wilson T, Zubair H, Xie L, Chambers E, et al. Developing a Food Exposure and Urine Sampling Strategy for Dietary Exposure Biomarker Validation in Free-Living Individuals. Molecular Nutrition & Food Research. 2019;63(14):1900062.
48. Boyle M, Tiniakos D, Schattenberg JM, Ratziu V, Bugianessi E, Petta S, et al. Performance of the PRO-C3 collagen neo-epitope biomarker in non-alcoholic fatty liver disease. JHEP Reports. 2019;1(3):188-98.
49. Eldridge SM, Chan CL, Campbell MJ, Bond CM, Hopewell S, Thabane L, et al. CONSORT 2010 statement: extension to randomised pilot and feasibility trials. BMJ. 2016;355:i5239.
50. Suárez M. Mediterranean Diet and Multi-Ingredient-Based Interventions for the Management of Non-Alcoholic Fatty Liver Disease. Nutrients. 2017;9:1052.
51. Penn L, Boeing H, Boushey CJ, Dragsted LO, Kaput J, Scalbert A, et al. Assessment of dietary intake: NuGO symposium report. Genes Nutr. 2010;5(3):205-13.
52. Younge JO, Kouwenhoven-Pasmooij TA, Freak-Poli R, Roos-Hesselink JW, Hunink MGM. Randomized study designs for lifestyle interventions: a tutorial. International Journal of Epidemiology. 2015;44(6):2006-19.
53. Harris JE, Raynor HA. Crossover Designs in Nutrition and Dietetics Research. Journal of the Academy of Nutrition and Dietetics. 2017;117(7):1023-30.
54. Keogh GF, Cooper GJS, Mulvey TB, McArdle BH, Coles GD, Monro JA, et al. Randomized controlled crossover study of the effect of a highly β-glucan–enriched barley on cardiovascular disease risk factors in mildly hypercholesterolemic men. The American Journal of Clinical Nutrition. 2003;78(4):711-8.
55. Favé G, Beckmann M, Lloyd AJ, Zhou S, Harold G, Lin W, et al. Development and validation of a standardized protocol to monitor human dietary exposure by metabolite fingerprinting of urine samples. Metabolomics. 2011;7(4):469-84.

Figure legends

Figure 1. Overview of the randomised controlled feasibility trial

Declarations

Authors' contributions

LH, JCM, and QMA conceived of the study; LH, SMcP, JCM, and QMA contributed to design of the study; All authors revised the manuscript for important intellectual content with JCM and QMA giving final approval of the version to be published.

Funding statement

This research was funded by the NIHR Newcastle Biomedical Research Centre (BRC) (grant number BRC-1215-20001). The NIHR Newcastle Biomedical Research Centre (BRC) is a partnership between Newcastle Hospitals NHS Foundation Trust and Newcastle University, funded by the National Institute for Health Research (NIHR). The views expressed are those of the authors and not necessarily those of the NIHR or the Department of Health and Social Care. QMA is a member of the LITMUS (Liver Investigation: Testing Marker Utility in Steatohepatitis) consortium funded by the Innovative Medicines Initiative (IMI2) Program of the European Union (Grant Agreement 777377).

Competing interests statement

The authors have no competing interests to declare that are relevant to the content of this article.

For peer review only - http://bmjopen.bmj.com/site/about/guidelines.xhtml

For peer review only

SMcP: Consultancy/speakers fees- Abbvie, Allergan, BMS, Gilead, Intercept, MSD, Novartis and Sequana. QMA: Research Grant Funding Abbvie, Allergan/Tobira, AstraZeneca, GlaxoSmithKline, Glympse Bio, Novartis Pharma AG, Pfizer Ltd., Vertex. Active Research Collaborations (including research collaborations supported through the EU IMI2 LITMUS Consortium*) Abbvie, Antaros Medical*, Allergan/Tobira*, AstraZeneca*, BMS*, Boehringer Ingelheim International GMBH*, Echosens*, Ellegaard Gottingen Minipigs AS*, Eli Lilly & Company Ltd.*, Exalenz Bioscience Ltd.*, Genfit SA*, Glympse Bio, GlaxoSmithKline, HistoIndex*, Intercept Pharma Europe Ltd.*, iXscient Ltd.*, Nordic Bioscience*, Novartis Pharma AG*, Novo Nordisk A/S*, One Way Liver Genomics SL*, Perspectum Diagnostics*, Pfizer Ltd.*, Resoundant*, Sanofi-Aventis Deutschland GMBH*, SomaLogic Inc.*, Takeda Pharmaceuticals International SA*. Consultancy 89Bio, Abbott Laboratories, Acuitas Medical, Allergan/Tobira, Altimmune, AstraZeneca, Axcella, Blade, BMS, BNN Cardio, Celgene, Cirius, CymaBay, EcoR1, E3Bio, Eli Lilly & Company Ltd., Galmed, Genentech, Genfit SA, Gilead, Grunthal, HistoIndex, Indalo, Imperial Innovations, Intercept Pharma Europe Ltd., Inventiva, IQVIA, Janssen, Madrigal, MedImmune, Metacrine, NewGene, NGMBio, North Sea Therapeutics, Novartis, Novo Nordisk A/S, Pfizer Ltd., Poxel, ProSciento, Raptor Pharma, Servier, Terns, Viking Therapeutics. Speaker Abbott Laboratories, Allergan/Tobira, BMS, Clinical Care Options, Falk, Fishawack, Genfit SA, Gilead, Integritas Communications, Kenes, MedScape. Royalties Elsevier Ltd (Davidson's Principles & Practice of Medicine textbook)

For peer review only

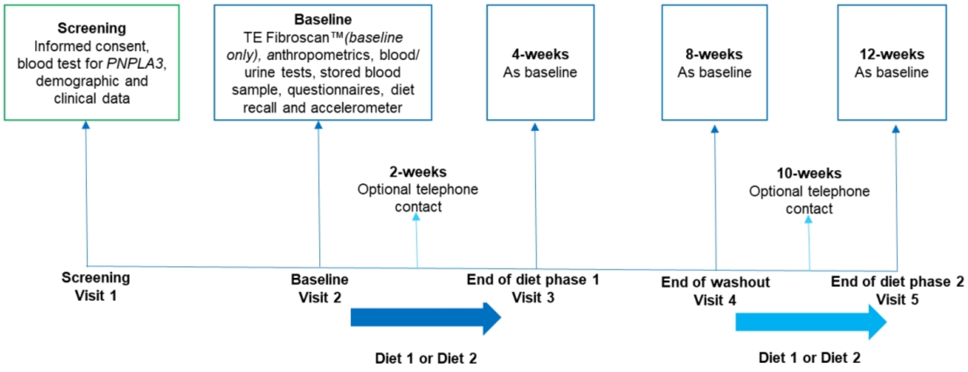

Figure 1. Overview of the randomised controlled feasibility trial

396x162mm (96 x 96 DPI)

Appendix

Table 1. Mediterranean Diet Meal Options

Meal (one serving per container)	Protein(g)	Carb(g)	Fat(g)	Calories
Beef Bolognese with vegetables and whole-wheat pasta	42	43	14	466
Romesco chicken with vegetables and whole-wheat pasta	38	45	17	485
Garlic and herb chicken with vegetable medley	36	27	16	396
Chicken, basil and tomato stir fry	36	40	18	466
Mediterranean falafels with vegetable medley and tahini	10	39	15	331
Cod on vegetable pasta with tomato and basil sauce	30	45	14	426
Halloumi cashew nut and broccoli curry	26	50	23	511
Tomato, black bean with cashews and quinoa	12	36	16	361
Tomato chickpea vegetable curry	14	38	13	353
Chipotle sweet potato chilli with spinach	20	65	6	394
Chicken salad with pesto pasta	36	30	15	399
Garlic and herb chicken salad	32	25	9	309
Tuna and cheese with pesto pasta salad	36	38	12	404
Feta, pine nut and pesto cous salad	16	40	17	377
Red pesto salmon with vegetable medley	24	40	26	490
Honey chilli salmon salad	23	25	20	313
Mozzarella, roasted tomato and pesto pasta salad	19	47	25	477
Grilled halloumi and roasted pepper salad	23	25	23	399
Falafel salad with tahini, avocado and sriracha in Mediterranean herb wrap	20	55	18	462
Chicken with broccoli cashew nut pesto pasta	38	39	17	461
Lentil dahl	23	75	5	437
Vegetable balls and tomato pesto sauce and pasta	12	56	16	416
Chickpea and vegetable curry with rice	23	70	16	516
Butternut, lentil and spinach	15	28	18	372

CONSORT 2010 checklist of information to include when reporting a pilot or feasibility trial*

Section/Topic	Item No	Checklist item	Reported on page No
**Title and abstract**			
	1a	Identification as a pilot or feasibility randomised trial in the title	Title page
	1b	Structured summary of pilot trial design, methods, results, and conclusions (for specific guidance see CONSORT abstract extension for pilot trials)	1
**Introduction**			
Background and objectives	2a	Scientific background and explanation of rationale for future definitive trial, and reasons for randomised pilot trial	3
	2b	Specific objectives or research questions for pilot trial	4
**Methods**			
Trial design	3a	Description of pilot trial design (such as parallel, factorial) including allocation ratio	4-5
	3b	Important changes to methods after pilot trial commencement (such as eligibility criteria), with reasons	N/A
Participants	4a	Eligibility criteria for participants	4-5
	4b	Settings and locations where the data were collected	4
	4c	How participants were identified and consented	4
Interventions	5	The interventions for each group with sufficient details to allow replication, including how and when they were actually administered	6-7
Outcomes	6a	Completely defined prespecified assessments or measurements to address each pilot trial objective specified in 2b, including how and when they were assessed	6-8
	6b	Any changes to pilot trial assessments or measurements after the pilot trial commenced, with reasons	N/A
	6c	If applicable, prespecified criteria used to judge whether, or how, to proceed with future definitive trial	9-10
Sample size	7a	Rationale for numbers in the pilot trial	4
	7b	When applicable, explanation of any interim analyses and stopping guidelines	N/A
Randomisation:			
Sequence generation	8a	Method used to generate the random allocation sequence	5
	8b	Type of randomisation(s); details of any restriction (such as blocking and block size)	N/A
Allocation concealment mechanism	9	Mechanism used to implement the random allocation sequence (such as sequentially numbered containers), describing any steps taken to conceal the sequence until interventions were assigned	N/A

For peer review only

Implementation	10	Who generated the random allocation sequence, who enrolled participants, and who assigned participants to interventions	N/A
Blinding	11a	If done, who was blinded after assignment to interventions (for example, participants, care providers, those assessing outcomes) and how	6
	11b	If relevant, description of the similarity of interventions	6-7
Statistical methods	12	Methods used to address each pilot trial objective whether qualitative or quantitative	8-9
**Results**			
Participant flow (a diagram is strongly recommended)	13a	For each group, the numbers of participants who were approached and/or assessed for eligibility, randomly assigned, received intended treatment, and were assessed for each objective	N/A
	13b	For each group, losses and exclusions after randomisation, together with reasons	N/A
Recruitment	14a	Dates defining the periods of recruitment and follow-up	11
	14b	Why the pilot trial ended or was stopped	N/A
Baseline data	15	A table showing baseline demographic and clinical characteristics for each group	N/A
Numbers analysed	16	For each objective, number of participants (denominator) included in each analysis. If relevant, these numbers should be by randomised group	N/A
Outcomes and estimation	17	For each objective, results including expressions of uncertainty (such as 95% confidence interval) for any estimates. If relevant, these results should be by randomised group	N/A
Ancillary analyses	18	Results of any other analyses performed that could be used to inform the future definitive trial	N/A
Harms	19	All important harms or unintended effects in each group (for specific guidance see CONSORT for harms)	N/A
	19a	If relevant, other important unintended consequences	N/A
**Discussion**			
Limitations	20	Pilot trial limitations, addressing sources of potential bias and remaining uncertainty about feasibility	10-11
Generalisability	21	Generalisability (applicability) of pilot trial methods and findings to future definitive trial and other studies	10-11
Interpretation	22	Interpretation consistent with pilot trial objectives and findings, balancing potential benefits and harms, and considering other relevant evidence	N/A
	22a	Implications for progression from pilot to future definitive trial, including any proposed amendments	N/A
**Other information**			
Registration	23	Registration number for pilot trial and name of trial registry	1
Protocol	24	Where the pilot trial protocol can be accessed, if available	N/A
Funding	25	Sources of funding and other support (such as supply of drugs), role of funders	14
	26	Ethical approval or approval by research review committee, confirmed with reference number	11

For peer review only

Citation: Eldridge SM, Chan CL, Campbell MJ, Bond CM, Hopewell S, Thabane L, et al. CONSORT 2010 statement: extension to randomised pilot and feasibility trials. BMJ. 2016;355.
*We strongly recommend reading this statement in conjunction with the CONSORT 2010, extension to randomised pilot and feasibility trials, Explanation and Elaboration for important clarifications on all the items. If relevant, we also recommend reading CONSORT extensions for cluster randomised trials, non-inferiority and equivalence trials, non-pharmacological treatments, herbal interventions, and pragmatic trials. Additional extensions are forthcoming: for those and for up to date references relevant to this checklist, see www.consort-statement.org.

For peer review only

BMJ Open

A Nutrigenetics-based Intervention Approach for Adults with Non-Alcoholic Fatty Liver Disease (NAFLD): Study Protocol for a Randomised Controlled Feasibility Trial

Journal:	*BMJ Open*
Manuscript ID	bmjopen-2020-045922.R1
Article Type:	Protocol
Date Submitted by the Author:	03-Feb-2021

Complete List of Authors:	Haigh, Laura; Newcastle Upon Tyne Hospitals NHS Trust, Regional Liver Unit; Newcastle University, Faculty of Medical Sciences<br>McPherson, Stuart; Newcastle Upon Tyne Hospitals NHS Trust, Regional Liver Unit; Newcastle University, Faculty of Medical Sciences<br>Mathers, John; Newcastle University, Human Nutrition Research Centre<br>Anstee, Quentin; Newcastle University, Faculty of Medical Sciences; Newcastle Upon Tyne Hospitals NHS Trust, Regional Liver Unit

**Primary Subject Heading**:	Gastroenterology and hepatology
Secondary Subject Heading:	Nutrition and metabolism
Keywords:	Hepatology < INTERNAL MEDICINE, NUTRITION & DIETETICS, THERAPEUTICS

SCHOLARONE™
Manuscripts

I, the Submitting Author has the right to grant and does grant on behalf of all authors of the Work (as defined in the below author licence), an exclusive licence and/or a non-exclusive licence for contributions from authors who are: i) UK Crown employees; ii) where BMJ has agreed a CC-BY licence shall apply, and/or iii) in accordance with the terms applicable for US Federal Government officers or employees acting as part of their official duties; on a worldwide, perpetual, irrevocable, royalty-free basis to BMJ Publishing Group Ltd ("BMJ") its licensees and where the relevant Journal is co-owned by BMJ to the co-owners of the Journal, to publish the Work in this journal and any other BMJ products and to exploit all rights, as set out in our licence.

The Submitting Author accepts and understands that any supply made under these terms is made by BMJ to the Submitting Author unless you are acting as an employee on behalf of your employer or a postgraduate student of an affiliated institution which is paying any applicable article publishing charge ("APC") for Open Access articles. Where the Submitting Author wishes to make the Work available on an Open Access basis (and intends to pay the relevant APC), the terms of reuse of such Open Access shall be governed by a Creative Commons licence – details of these licences and which Creative Commons licence will apply to this Work are set out in our licence referred to above.

Other than as permitted in any relevant BMJ Author's Self Archiving Policies, I confirm this Work has not been accepted for publication elsewhere, is not being considered for publication elsewhere and does not duplicate material already published. I confirm all authors consent to publication of this Work and authorise the granting of this licence.

For peer review only

A Nutrigenetics-based Intervention Approach for Adults with Non-Alcoholic Fatty Liver Disease (NAFLD): Study Protocol for a Randomised Controlled Feasibility Trial

[1,2,3]Laura Haigh, [1,2] Stuart McPherson, [3] John C Mathers*, [1,2] Quentin M Anstee*

*Joint Senior Authors

Author affiliations

[1]Regional Liver Unit, Newcastle upon Tyne Hospitals NHS Foundation Trust, Newcastle upon Tyne, UK.

[2] Translational and Clinical Research Institute, Faculty of Medical Sciences, Newcastle University, Newcastle upon Tyne, UK.

[3] Human Nutrition Research Centre, Population Health Sciences Institute, Faculty of Medical Sciences, Newcastle University, Newcastle upon Tyne, UK.

Correspondence: Laura Haigh, Regional Liver Unit, Newcastle upon Tyne Hospitals NHS Foundation Trust, Newcastle upon Tyne, UK. NE7 7DN. Email: l.haigh3@newcastle.ac.uk Tel: 0191 2231568 Fax: 0191 208 0723

Keywords: Non-alcoholic Fatty Liver Disease; Nutrigenetics; Metabolomics; Mediterranean diet

Word count: 3505

For peer review only

Abstract

Introduction: Lifestyle interventions targeting weight loss and improved dietary patterns are the recommended treatment for non-alcoholic fatty liver disease (NAFLD). However, the effectiveness of current established diet therapies is sub-optimal. The *PNPLA3* gene modifies disease outcome and hepatic lipid handling, but the role of *PNPLA3* variants in modulating responsiveness to different diet therapies is unknown.

Methods and analysis: This project aims to assess the feasibility of conducting a genotype-driven randomised controlled trial (RCT) investigating the differential response to a Mediterranean Diet (MD) intervention of NAFLD patients according to genotype for the rs738409 (I148M) variant of *PNPLA3*. A single centre randomised controlled feasibility trial will be undertaken. We will recruit 60 adults with NAFLD from a tertiary hepatology centre in England. In a cross-over design, participants will undertake Diet 1 (MD) and Diet 2 (control) for 4-weeks, in random order (1:1 allocation), separated by a 4-weeks washout period. Participants will complete one-to-one diet and lifestyle consultations at baseline, end of diet phase 1, end of washout and end of diet phase 2. Participants will be advised to maintain baseline levels of physical activity and body weight. The primary outcome is the acceptability and feasibility of the intervention protocol. Secondary outcomes include exploratory assessment of liver fibrosis biomarkers and lipid biomarkers.

Ethics and dissemination: Ethical approval was granted by East of Scotland Research Ethics Service REC 1 (19/ES/0112). Results will be disseminated through peer-reviewed journals and presented at local, national and international meetings and conferences. The findings of this trial will lay the foundation for a future definitive RCT by informing trial design and optimising the intervention diets, instruments and procedures.

Clinical trial registration: ISRCTN93410321

For peer review only - http://bmjopen.bmj.com/site/about/guidelines.xhtml

Article Summary

Strengths and limitations of this study

- The main strength of this trial is the use of explicit feasibility and acceptability criteria as primary outcomes.
- A unique feature of this trial is the use of metabolomics biomarkers to: i) capture diet-mediated effects on lipid metabolism and ii) provide objective assessment of dietary intake.
- In the definitive RCT that is expected to follow this feasibility trial, the use of a cross-over design will facilitate more precise comparisons between intervention/control diets on a within-participant basis.
- The effectiveness of washout to return outcome variables to baseline will be evaluated.
- The MD intervention has been systematically developed and informed by research evidence and patient and public feedback.
- This trial will generate qualitative and quantitative information to establish the feasibility and acceptability of the protocol.

For peer review only

For peer review only - http://bmjopen.bmj.com/site/about/guidelines.xhtml

Introduction

Non-alcoholic fatty liver disease (NAFLD) affects 25% of the global population (1, 2) and is a spectrum spanning steatosis (non-alcoholic fatty liver, NAFL), through steatosis plus inflammation, hepatocyte damage and progressive fibrosis (non-alcoholic steatohepatitis, NASH), that may potentially develop into cirrhosis and hepatocellular carcinoma (3-5). NAFLD is strongly associated with obesity and cardio-metabolic disease (2, 6).

NAFLD attributed rates of advanced liver disease and transplantation are increasing, but effective pharmacotherapy is unavailable (2, 3). A major challenge is the substantial inter-individual variation in NAFLD susceptibility, progression and outcome, due partly to gene-environment interactions (2, 3). The patatin-like phospholipase domain containing 3 *(PNPLA3)* rs738409 single nucleotide polymorphism is a common modifier of disease outcome and its impact is amplified by adiposity (7-10). This additive effect poses considerable concern, as patients with NAFLD tend to consume excess calories as a consequence of poor-quality diets and sedentary lifestyles (11-13).

The PNPLA3 protein has lipase activity and regulates lipid droplets in hepatocytes and hepatic stellate cells (14). Several studies have contributed to understanding of its function in hepatic lipid handling (15). Numerous genome-wide association studies (GWAS) have shown its association with the entire disease spectrum, which has been confirmed in various populations (8, 10, 12, 16, 17). Therefore, among the identified NAFLD-related genes, *PNPLA3* has the most potential as a therapeutic target for NAFLD (8, 10, 12).

The main treatment recommendations for NAFLD are lifestyle interventions targeting weight loss and improved dietary patterns (4, 5). The Mediterranean diet (MD) reduces steatosis, improves liver biochemistry and cardio-metabolic dysfunction, with or without weight loss and is the most recommended dietary pattern in NAFLD (5, 18-23). There is strong and consistent evidence that the MD, has beneficial effects on NAFLD associated conditions such as Type 2 Diabetes and the metabolic syndrome(24). However, there is sub-optimal response to current diet therapies in NAFLD and more effective approaches for enhancing adherence to diet therapies is needed (11).

Personalised nutrition approaches that use information on individual participant characteristics to tailor the dietary intervention may improve the sub-optimal response to current diet therapies (25). Differences in gene sequence can alter the activity of encoded proteins and affect the response of individuals to dietary components (26). Emergent research has shown nutritional regulation of *PNPLA3* (15, 26) so that patients with NAFLD carrying the *PNPLA3* risk allele might benefit more from weight loss but less from omega-3 supplementation (26-29). Diet lifestyle modification is more effective in decreasing liver steatosis in *PNPLA3 I148M* carriers than in non-carriers (29, 30). Liver fat content is influenced by the interaction between *PNPLA3* variants and high carbohydrate intake, specifically sugar (31). A hypocaloric low-carbohydrate diet induced greater hepatic fat reduction in carriers homozygous for the rs738409 G allele in

For peer review only - http://bmjopen.bmj.com/site/about/guidelines.xhtml

the *PNPLA3* gene compared with carriers of the rs738409 C allele, irrespective of weight loss (32). However, the role of *PNPLA3* variants in influencing responsiveness to different diet therapies is poorly understood.

This project aims to determine whether it is feasible to conduct a RCT to investigate the impact of *PNPLA3* carriage on responsiveness to MD and NAFLD severity and to provide preliminary data to inform the development of a definitive RCT.

Methods and analysis

Study design and setting

This trial is a single centre, randomised controlled feasibility trial. In a cross-over design, participants will be randomised to either Diet 1 (MD) or Diet 2 (control) for 4-weeks, in random order, separated by a 4-weeks washout period. All study visits will be conducted in the outpatient hepatology services, in The Newcastle upon Tyne Hospitals NHS Foundation Trust, (NuTH) United Kingdom. An overview of the study design is provided in Figure 1.

Study objectives

The primary objective is to determine whether the protocol for a future definitive RCT is acceptable and feasible.

We will:

- Determine the feasibility of recruitment and retention.
- Determine the acceptability of the diets, instruments and procedures.
- Evaluate adherence to, and completion of, the diets and procedures.
- Evaluate implementation fidelity and how practicable it is to deliver the protocol in a clinical setting.
- Estimate outcome variability.

Secondary objectives include collection of preliminary exploratory data on liver fibrosis biomarkers and lipid biomarkers, to determine if *PNPLA3* carriage influences response to diet treatments. This mixed methods feasibility trial will generate qualitative and quantitative information about feasibility and acceptability to inform a future definitive RCT.

Sampling and recruitment

A recruitment target of 60 individuals with either imaging or histological evidence of NAFLD was established in accordance with published guidance (33). This guidance suggests a sample of 30 individuals or greater, is sufficient to estimate a parameter with the necessary degree of precision (33). Adults with NAFLD will be recruited from a tertiary hepatology centre in NuTH, which covers a population of approximately 3million in Northern United Kingdom. Potential participants meeting the inclusion and exclusion criteria will be identified from NuTH hepatology clinic lists and electronic records by members of the research and clinical care team. Potential participants will be approached during an appointment in liver outpatients or through screening of clinical notes and invited to participate in the study. The research team

For peer review only - http://bmjopen.bmj.com/site/about/guidelines.xhtml

will contact patients after 48 hours to answer any queries. Enrolment will follow the receipt of full and written informed consent and successful screening. Recruitment and intervention delivery are anticipated to take place over 12 months.

Inclusion and exclusion criteria

Participant eligibility criteria are detailed in Table 1. We have chosen minimal exclusions to reflect the target population for a subsequent definitive RCT.

Table 1. Patient eligibility criteria

Inclusion criteria
• 18-80 years old with NAFLD (confirmed on liver biopsy or by clinical diagnosis with imaging evidence of steatosis).
• Weekly alcohol consumption <14(women)/ <21(men) units in the last 24months.
• Weight stable (+/-5%) for previous 3 months.
• Capacity to provide informed consent.
• Ability to write and converse in English without assistance of an interpreter.
Exclusion criteria
• All cancers within 5 years (except squamous cell carcinoma).
• Evidence of co-existent liver disease/ presence of secondary causes of NAFLD (except Gilbert's syndrome).
• Decompensated NASH-cirrhosis (Child Pugh >6).
• Uncontrolled psychiatric disorder (e.g. acute psychosis).
• Uncontrolled medical condition (e.g. HbA1c ≥80mmol/l or acute coronary event or stroke within 12 months).
• Active eating disorder.
• Active substance misuse.
• Other prescribed dietary regimens, food intolerances and/or food allergies.
• Mediterranean diet score (MEDAS) >8 (high MD consumption).
• Previous weight loss surgery.
• Taking anti-obesity medications and/ or engaged in structured, multi-component weight management interventions (specialist, community or commercial providers).
• Insulin use.
• Pregnancy/ lactation.

Randomisation and allocation strategy: Eligible participants will be allocated in a 1:1 ratio to receive either Diet 1 or Diet 2 first using computer generated randomisation. Pre-randomisation genotyping for specific genetic variants (*PNPLA3 rs738409*) will be conducted using blood samples collected at screening. Interim analysis of the genotype distribution amongst recruited cohort will be conducted at intervals as

For peer review only - http://bmjopen.bmj.com/site/about/guidelines.xhtml

recruitment proceeds (e.g. after one third and two-thirds have been randomised); randomisation will then be stratified by *PNPLA3* status at baseline to ensure a balanced recruitment for *PNPLA3 rs738409* genetic status (wildtype, heterozygote, homozygote).

The major allele frequency for *PNPLA3 rs738409* is approximately 0.25 in the UK general population (34) and the minor allele frequency (MAF) is greater in those with NAFLD (17). Accordingly, we expect to reach the recruitment target for wild-type cases sooner than for carriers of the genetic variant and so some patients with a specific genotype may not randomised if sufficient cases of a given genotype are already enrolled. Those discharged from the study will be offered a one-to-one nutrition education and counselling consultation with a dietitian. It will not be possible to blind participants, clinicians or research investigators to which diet each participant is on in each study period.

Trial procedures

The trial procedures are outlined in Table 2. At an initial screening appointment, demographic, clinical, dietary and lifestyle data will be collected to determine patient eligibility and a blood sample will be taken for *PNPLA3 rs738409* genotyping. In addition, at baseline, liver steatosis will be assessed using Controlled Attenuation Parameter (CAP) measured contemporaneously with liver stiffness by Transient Elastography, Fibroscan®.

Enrolled participants will complete one-to-one diet and lifestyle consultations at baseline, end of diet phase 1 (4-weeks), end of washout (8-weeks) and end of diet phase 2 (12-weeks). At each of these time-points, the following measures will be taken; anthropometry and body composition; blood biochemistry; dietary intake, urine samples and physical activity. The clinical status and medication consumption of participants will be checked at each time-point. Patient-reported outcome data will be captured at the end of each diet phase (4-weeks and 12-weeks).

Dietary intervention and control treatments

The experimental Diet 1 is a Mediterranean-style diet (MD) based on the traditional MD and MD pyramid (35). The MD is characterised by a high quantity of plant-based foods, unrefined cereals, fruit and vegetables, olive oil, and nuts; eating white meat, fish and legumes in moderation; restricting red and processed meats, and sweets; and drinking red wine moderately (36). The diet was designed to be easy to follow over 4 weeks, informed by research evidence (37) and the findings from our earlier pilot study with patients and the public that explored barriers and facilitators to adoption of a MD intervention (38). They highlighted the importance of reducing the burden of dietary changes, diet supporters in the household, regular nutritional counselling and preference for face-to-face contacts. These ideas have been incorporated into the final design.

To reduce participant burden and facilitate changes in food environment, some intervention foods will be supplied as pre-packaged ready meals (FreshPrepare©) and extra virgin olive oil 750ml (Filippo Berio©).

For peer review only - http://bmjopen.bmj.com/site/about/guidelines.xhtml

Ten pre-packaged ready meals will be home delivered weekly, taken as two main meals per day (lunch and evening meal) for five days. The meals can be ordered online, and examples of the available options are detailed in Appendix Table 1. The provision of these intervention foods will assist in standardising food consumption and minimise variability in dietary intake.

To enhance MD adoption, nutrition counselling and education will be provided one-to-one to participants during visits, by a dietitian. This will include advice on the selection and preparation of appropriate meals, snacks and drinks. The consultation incorporates the 'model and process for nutrition and dietetic practice', (39) and involves participant discussion. Personalisation of the diet for specific personal and sociocultural preferences will be provided.

A patient information booklet has been designed to explain NAFLD, the principles of MD, and how it can be successfully followed. This will be given to participants as evidence-based written material from a credible source. Behaviour change techniques will be utilised to increase intervention effectiveness. These include barrier identification, problem solving, goal setting, and action planning; social support; and instruction on how to perform a behaviour and behavioural practice (40-42).

Diet 2 (control) will involve counselling participants to consume their habitual diet. During washout participants will be asked to return to their habitual diet. Participants will be asked to maintain baseline levels of physical activity and body weight (+/-3%) throughout the trial duration. The dietitian will contact participants by telephone mid-way through each diet phase to review progress, provide additional counselling and answer any queries.

For peer review

Table 2. Schedule of study measures

Variable	Instrument	Timepoint				
		Screening	Baseline	End of diet phase 1	End of washout	End of diet phase 2
Inclusion/ exclusion criteria		×				
Informed consent		×				
Demographic data and medical history	Self-report and clinical records	×				
Genotyping	Venous blood sample	×				
Anthropometrics: weight, height, waist and hip circumference			×	×	×	×
Whole body composition	Bioelectrical Impedance Analysis		×	×	×	×
Cardio-metabolic measures: glucose,						

insulin, HbA1c, lipid profile and blood pressure	Fasting venous blood samples		×	×	×	×
Liver function: LFTs, Ferritin, FBC, CRP, lipid biomarkers	Fasting venous blood samples		×	×	×	×
Liver steatosis: CAP	TE Fibroscan™		×			
Liver fibrosis:						
Liver stiffness, and	TE Fibroscan™		×			
PRO-C3	Fasting venous blood samples		×	×	×	×
Patient-reported outcomes	EQ5D, CLDQ-NASH and NASH-CHECK			×		×
Physical activity	Accelerometer		×	×	×	×
Dietary intake:						
dietary biomarkers, diet	Urine samples		×	×	×	×
recall and MD	INTAKE24		×	×	×	×
questionnaire	MEDAS		×	×	×	×

Abbreviations: HbA1c, glycated haemoglobin; LFTs, liver function tests; FBC, full blood count; CRP, C-Reactive Protein; CAP, controlled attenuation parameter; TE, transient elastography; PRO-C3, N-protease cleaved PIIINP neo-epitope; EQ-5D, euroqol five dimension scale; CLDQ-NASH, chronic liver disease questionnaire for non-alcoholic steatohepatitis; MEDAS, Mediterranean diet assessment score.

Outcome assessment and process evaluation: The following criteria will be assessed.

- The feasibility of recruitment and retention

The consent rate (the number of eligible participants who consent divided by the total number who are eligible and who are invited to participate), the recruitment rate (the number of participants recruited per month), and the retention rate (the number of participants who complete follow-up data collection divided by the total number randomised) will be assessed using a trial log between baseline and end of study.

- The acceptability of the diets, instruments and procedures

Patient-reported outcome data will be used to identify impact of diet treatments and trial procedures on quality of life using the PROs 'EQ5D', 'CLDQ-NASH' and 'NASH-CHECK' (43-45). At 12 weeks, open-response questions will be used to capture participant perceptions of: i) the randomisation procedure, ii) the, acceptability of the MD intervention and iii) the components of the measurement protocol. These data will be recorded (Appendix 2), and thematic analyses performed.

- Adherence to, and completion of, the diets and procedures

Participant adherence to trial procedures will be assessed by tracking the number of completed visits, and the completeness of data collection will be assessed using the trial log between baseline and 12 weeks.

For peer review only - http://bmjopen.bmj.com/site/about/guidelines.xhtml

Diet adherence will be assessed in self-report measures; Mediterranean diet assessment score (MEDAS) is based on a small number of foods measured in servings/d or servings/week. Scores range between 0-14, and can be categorised as low, moderate or high consumption (<5, 6-9 and >10 points, respectively) (46). Dietary intake will be quantified using INTAKE24, an open-source computerised dietary recall system based on multiple-pass 24-hour recall (47, 48). INTAKE24 data will be converted to MD scores and Dietary Inflammatory Index™ will be calculated (49).

In addition, dietary biomarkers will be quantified in urine to provide objective measures of dietary intake, without self-report bias (50). Three spot urine samples will be collected at the beginning and end of each diet phase on non-consecutive days, including one weekend day, to provide estimates of habitual dietary intake. The three urine samples from each time-point will be pooled and analysed using Ultra High-Performance Liquid Chromatography (UHPLC) (51).

- Implementation fidelity and how practicable protocol processes are to deliver in a clinical setting

Data collection burden will be measured as the time taken to administer protocol processes. Participant processing time is the number of days from initial contact to enrolment. Both will be assessed using the trial log between baseline and end of study. To assess integrity and fidelity, a trial protocol checklist will be monitored with missing, incomplete or unreliable data recorded between baseline and end of study.

- Preliminary exploratory data on liver fibrosis biomarkers and lipid biomarkers

In collaboration with partners in the EU IMI2-funded LITMUS biomarkers consortium (https://litmus-project.eu/), plasma will be analysed to characterise changes in the lipidome and metabolome to help elucidate mechanisms underlying specific changes in lipid metabolism. Plasma PRO-C3 concentration, as a marker of fibrogenesis, will be measured using competitive ELISAs (52).

The data from this feasibility trial will be reported as descriptive summaries. Sample characteristics including age, gender, ethnicity, medical history and disease sub-classification and severity will be presented. Although the trial is not powered to detect significant changes in clinical and lifestyle outcomes, key variables of interest (outlined in schedule of assessments) will be monitored and any preliminary changes reported. These data will determine the feasibility of testing components and enable the statistical power calculations for a subsequent RCT. The balance of the groups after randomisation will be explored. Descriptive statistics ((mean, standard deviation (SD) and counts (percentage)) will summarise continuous and categorical data as appropriate. No imputation of missing data will be undertaken.

Success criteria

The feasibility indicators are binary (successful or unsuccessful). "Successful" would indicate the protocol is sufficiently robust to advance to a fully powered definitive RCT, whilst "unsuccessful" indicates that protocol changes are required (53).

For peer review only - http://bmjopen.bmj.com/site/about/guidelines.xhtml

- The acceptability of diets, instruments and procedures.
- Consent rate (25% of individuals consenting).
- Recruitment rate (target 60 individuals/9months) (acceptable 45 individuals/9months).
- Retention rate (target 45 individuals/3months) (acceptable 36 individuals/3months).
- Participant adherence (75% completed visits/data collection).
- Data collection burden (target >75% of individuals≤1.5h) (acceptable >75% of individuals<2hr).
- Participant processing time (mean time <14days between initial contact to enrolment).
- Trial protocol administration (<10% deviation from checklist).

Patient and public involvement

The early stages of the research process involving the preparation of the research proposal were supported by a national liver patient support group (LIVErNORTH). LIVErNORTH collaborate on the joint production of the patient information booklet, which is used in one-to-one diet and lifestyle consultations. To enhance the development of the MD intervention the findings from an earlier pilot study (54) and research evidence were discussed with a patient panel (APEX) as well as patients attending clinical services in NuTH. APEX advised on the patient experience and assessed the burden of the trial instruments and procedures. This patient and public feedback was integrated into the final design. There are plans in the future to involve patients and the public as the trial progresses, guiding the dissemination of trial results to participants and relevant wider patient communities.

Discussion

There is an urgent need to identify potential therapeutic interventions that can prevent NAFLD progression and induce regression (11). Lifestyle interventions to induce weight loss and improve dietary lifestyle patterns are the mainstay of NAFLD treatment (4, 5). However, diet lifestyle targets are often difficult to achieve in practice and definitive data are needed on the optimal strategies to enhance adherence to diet treatments (11). Stratified and targeted diet therapies may be an advance on the relatively ineffective 'one-size fits all' treatments. To that end, there is a need to explore the underlying mechanisms through which genotype influences responses to dietary components, and the subsequent effects on NAFLD.

The primary objective of this trial is to determine the acceptability and feasibility of a nutrigenetic approach for adults with NAFLD. This trial adopts a mixed methods approach designed to establish whether the protocol is sufficiently robust and to inform any necessary refinements to diets, instruments and procedures. In addition to the primary outcomes, the trial will provide data on variability of secondary outcomes for power calculations to inform the design of a subsequent RCT.

A unique feature of this trial is the use of metabolomics biomarkers to: i) capture diet-mediated effects on lipid metabolism and ii) provide objective assessment of dietary intake. Metabolomics approaches provide novel insights into complex disease traits and provide a powerful tool to predict and monitor responsiveness to diet treatments for NAFLD (55). The application of 'omics' could support the design of

For peer review only - http://bmjopen.bmj.com/site/about/guidelines.xhtml

innovative and effective diet therapies across the full NAFLD spectrum, and to understand their mechanisms (55). The use of self-reported dietary intake assessment methods presents significant challenges both in research and clinical practice (56) and some of these challenges can be overcome using urine-based biomarker approaches (50). Thus, we will combine self-reported data with the quantification of urinary dietary biomarkers to overcome this weakness.

The main strength of this trial is the use of explicit feasibility and acceptability criteria as primary outcomes. Patient-reported outcome measures and open-response questions will be especially important to identify impact of diet treatments and trial procedures on quality of life and individuals' perceptions of trial participation. In addition, the use of a cross-over design will facilitate more precise comparisons between intervention/control diets on a within-participant basis (57). This design is favoured in short-term trials of long-term conditions with intermediate outcomes (57). Nevertheless, a limitation of this design is the possibility of carry-over effects from one experimental period to the next. We have addressed this issue by including a 4-week washout period. The required duration of washout period is influenced by the nature and duration of the intervention but, in nutritional cross-over studies, 2-4 weeks is often sufficient (58, 59). This is confirmed by studies of metabolomics biomarkers in urine which show that these respond rapidly to dietary change (60). Importantly, we will evaluate the effectiveness of washout to return outcome variables to baseline. Additionally, participants will be supplied with intervention foods and counselled to return to habitual dietary intake during washout. Advice will be given to maintain baseline levels of physical activity and body weight (+/-3%) throughout. The intervention period (4-weeks) is relatively short and is unlikely to reveal the full effects of the dietary intervention on markers of liver health. Previous research has found that brief and short-term interventions effectively modified MD adoption and maintenance up to 12 months in non-Mediterranean countries (38, 61-63) and that, with appropriate support, dietary change can be sustained for up to 4 years in Spain (64). However, this study is designed, primarily, to provide information on the acceptability and feasibility of the study protocol. Finally, the MD intervention in this trial has been systematically developed and informed by research evidence and patient and public feedback.

This trial will assess the feasibility of conducting a genotype-driven RCT investigating the differential response to a MD intervention of patients according to genotype for the rs738409 (I148M) variant of *PNPLA3*. The findings of this trial will lay the foundation for a future definitive RCT by informing trial design and optimising the intervention diets, instruments and procedures. In the longer run, the outcomes of this research programme may lead to fewer NAFLD patients who need intensified medical treatment, reducing cost burden, and premature mortality and morbidity.

Ethics and dissemination

Ethical approval was granted by East of Scotland Research Ethics Service REC 1 (19/ES/0112). NHS Research and Development approval was granted by the Newcastle upon Tyne Hospitals NHS Foundation Trust (R&D8985). This trial will be conducted to a high standard in accordance with the protocol, the principles of good clinical practice (GCP), relevant regulations, guidelines and with regard to patient safety

For peer review only - http://bmjopen.bmj.com/site/about/guidelines.xhtml

and welfare. The findings of this trial will be submitted for publication in peer-reviewed journals and presented at local, national and international meetings and conferences. A lay summary of results will be available for all study participants.

Trial status

The first participant was enrolled on the 11th February 2020 with recruitment expected to be completed by the 31st March 2021. The recruitment period has been extended to mitigate the potential impact of COVID-19 on the research plans.

References

1. Younossi ZM, Koenig AB, Abdelatif D, Fazel Y, Henry L, Wymer M. Global epidemiology of nonalcoholic fatty liver disease-Meta-analytic assessment of prevalence, incidence, and outcomes. Hepatology. 2016;64(1):73-84.
2. Younossi Z, Tacke F, Arrese M, Chander Sharma B, Mostafa I, Bugianesi E, et al. Global Perspectives on Nonalcoholic Fatty Liver Disease and Nonalcoholic Steatohepatitis. Hepatology. 2019;69(6):2672-82.
3. Anstee QM, Targher G, Day CP. Progression of NAFLD to diabetes mellitus, cardiovascular disease or cirrhosis. Nat Rev Gastroenterol Hepatol. 2013;10(6):330-44.
4. Chalasani N, Younossi Z, Lavine J, Mae Diehl A, Brunt E, Cusi K, et al. The diagnosis and management of non-alcoholic fatty liver disease: Practice guideline by the American Gastroenterological Association, American Association for the Study of Liver Diseases, and American College of Gastroenterology. Gastroenterology. 2012;143(2):503.
5. European Association for the Study of the Liver (EASL), European Association for the Study of Diabetes (EASD), European Association for the Study of Obesity (EASO). EASL–EASD–EASO Clinical Practice Guidelines for the management of non-alcoholic fatty liver disease. Hepatology. 2016;64:1388–402.
6. Younossi Z, Anstee QM, Marietti M, Hardy T, Henry L, Eslam M, et al. Global burden of NAFLD and NASH: trends, predictions, risk factors and prevention. Nat Rev Gastroenterol Hepatol. 2016;15(1):11-20.
7. Anstee QM, Day CP. The Genetics of Nonalcoholic Fatty Liver Disease: Spotlight on PNPLA3 and TM6SF2. Semin Liver Dis. 2015;35(3):270-90.
8. Liu YL, Patman GL, Leathart JB, Piguet AC, Burt AD, Dufour JF, et al. Carriage of the PNPLA3 rs738409 C >G polymorphism confers an increased risk of non-alcoholic fatty liver disease associated hepatocellular carcinoma. J Hepatol. 2014;61(1):75-81.
9. Stender S, Kozlitina J, Nordestgaard BG, Tybjaerg-Hansen A, Hobbs HH, Cohen JC. Adiposity amplifies the genetic risk of fatty liver disease conferred by multiple loci. Nat Genet. 2017;49(6):842-7.
10. Mann JP, Anstee QM. NAFLD: PNPLA3 and obesity: a synergistic relationship in NAFLD. Nat Rev Gastroenterol Hepatol. 2017;14(9):506-7.
11. Romero-Gomez M, Zelber-Sagi S, Trenell M. Treatment of NAFLD with diet, physical activity and exercise. Journal of Hepatology. 2017;67(4):829-46.
12. Dong XC. PNPLA3-A Potential Therapeutic Target for Personalized Treatment of Chronic Liver Disease. Frontiers in medicine. 2019;6:304-.
13. Gerber L, Otgonsuren M, Mishra A, Escheik C, Birerdinc A, Stepanova M, et al. Non-alcoholic fatty liver disease (NAFLD) is associated with low level of physical activity: a population-based study. Aliment Pharmacol Ther. 2012;36:772–81.
14. Pingitore P, Romeo S. The role of PNPLA3 in health and disease. Biochimica et Biophysica Acta (BBA) - Molecular and Cell Biology of Lipids. 2019;1864(6):900-6.
15. Basu Ray S. PNPLA3-I148M: a problem of plenty in non-alcoholic fatty liver disease. Adipocyte. 2019;8(1):201-8.
16. Valenti L, Al-Serri A, Daly AK, Galmozzi E, Rametta R, Dongiovanni P, et al. Homozygosity for the patatin-like phospholipase-3/adiponutrin I148M polymorphism influences liver fibrosis in patients with nonalcoholic fatty liver disease. Hepatology. 2010;51(4):1209-17.
17. Anstee QM, Darlay R, Cockell S, Meroni M, Govaere O, Tiniakos D, et al. Genome-wide association study of non-alcoholic fatty liver and steatohepatitis in a histologically-characterised cohort. Journal of Hepatology. 2020.

For peer review only - http://bmjopen.bmj.com/site/about/guidelines.xhtml

18. Ryan MC, Itsiopoulos C, Thodis T, Ward G, Trost N, Hofferberth S, et al. The Mediterranean diet improves hepatic steatosis and insulin sensitivity in individuals with non-alcoholic fatty liver disease. Journal of hepatology. 2013;59(1):138-43.

19. Katsagoni CN, Egkomiti A, Papageorgiou M, Ioannidou P, Fragopoulou E, Papatheodoridis G, et al. Improvement in liver function after an intervention based on the Mediterranean diet in patients with non alcoholic fatty liver disease (NAFLD). Clinical Nutrition ESPEN. 2016;13:e57.

20. Gelli C, Tarocchi M, Abenavoli L, Di Renzo L, Galli A, De Lorenzo A. Effect of a counseling-supported treatment with the Mediterranean diet and physical activity on the severity of the non-alcoholic fatty liver disease. World J Gastroenterol. 2017;23(17):3150-62.

21. Aller R, Izaola O, de la Fuente B, de Luis D. Mediterranean diet is associated with liver histology in patients with non alcoholic fatty liver disease. Nutricion Hospitalaria. 2015;32(6):2518-24.

22. Shai I, Schwarzfuchs D, Henkin Y, Shahar DR, Witkow S, Greenberg I, et al. Weight Loss with a Low-Carbohydrate, Mediterranean, or Low-Fat Diet. New England Journal of Medicine. 2008;359(3):229-41.

23. Properzi C, O'Sullivan TA, Sherriff JL, Ching HL, Jeffrey GP, Buckley RF, et al. Ad Libitum Mediterranean and Low-Fat Diets Both Significantly Reduce Hepatic Steatosis: A Randomized Controlled Trial. Hepatology. 2018;68(5):1741-54.

24. Salas-Salvadó J, Guasch-Ferré M, Lee CH, Estruch R, Clish CB, Ros E. Protective Effects of the Mediterranean Diet on Type 2 Diabetes and Metabolic Syndrome. J Nutr. 2015;146(4):920s-7s.

25. Ordovas JM, Ferguson LR, Tai ES, Mathers JC. Personalised nutrition and health. Bmj. 2018;361:bmj.k2173.

26. Dongiovanni P, Valenti L. A Nutrigenomic Approach to Non-Alcoholic Fatty Liver Disease. Int J Mol Sci. 2017;18(7):1534.

27. Scorletti E, West AL, Bhatia L, Hoile SP, McCormick KG, Burdge GC, et al. Treating liver fat and serum triglyceride levels in NAFLD, effects of PNPLA3 and TM6SF2 genotypes: Results from the WELCOME trial. J Hepatol. 2015;63(6):1476-83.

28. Kalafati I, al. e. Genetics of Nonalcoholic Fatty Liver Disease: Role of Diet as a modifying factor Clin Nut. 2014;3:223-32.

29. Shen J, Wong GL, Chan HL, Chan RS, Chan HY, Chu WC, et al. PNPLA3 gene polymorphism and response to lifestyle modification in patients with nonalcoholic fatty liver disease. J Gastroenterol Hepatol. 2015;30(1):139-46.

30. Krawczyk M, Stachowska E, Milkiewicz P, Lammert F, Milkiewicz M. Reduction of Caloric Intake Might Override the Prosteatotic Effects of the PNPLA3 p.I148M and TM6SF2 p.E167K Variants in Patients with Fatty Liver: Ultrasound-Based Prospective Study. Digestion. 2016;93(2):139-48.

31. Davis JN, Le KA, Walker RW, Vikman S, Metz DS, Weigensberg MJ, et al. Increased hepatic fat in overweight Hispanic youth influenced by interaction between genetic variation in PNPLA3 and high dietary carbohydrate and sugar consumption. Am J Clin Nutr. 2010;92(6):1522-7.

32. Sevastianova K, Kotronen A, Gastaldelli A, Perttila J, Hakkarainen A, Lundbom J, et al. Genetic variation in PNPLA3 (adiponutrin) confers sensitivity to weight loss-induced decrease in liver fat in humans. Am J Clin Nutr. 2011;94(1):104-11.

33. Lancaster GA, Dodd S, Williamson PR. Design and analysis of pilot studies: recommendations for good practice. Journal of Evaluation in Clinical Practice. 2004;10(2):307-12.

34. Ensembl. Variant: rs738409 SNP, 2020, April, [Available from: https://apr2020.archive.ensembl.org/Homo_sapiens/Variation/Population?db=core;r=22:43928347-43929347;v=rs738409;vdb=variation;vf=88266706.

35. Bach-Faig A, Berry EM, Lairon D, Reguant J, Trichopoulou A, Dernini S, et al. Mediterranean diet pyramid today. Science and cultural updates. Public Health Nutrition. 2011;14(12A):2274-84.

36. Anania C, Perla FM, Olivero F, Pacifico L, Chiesa C. Mediterranean diet and nonalcoholic fatty liver disease. World J Gastroenterol. 2018;24(19):2083-94.

37. Wareham N. Fenland Study investigates interaction between genetic and lifestyle factors in determining obesity, diabetes and related metabolic disorders which present a considerable public health concern. . ISRCTN register 2013.

38. Haigh L, Bremner S, Houghton D, Henderson E, Avery L, Hardy T, et al. Barriers and Facilitators to Mediterranean Diet Adoption by Patients With Nonalcoholic Fatty Liver Disease in Northern Europe. Clinical Gastroenterology and Hepatology. 2019;17(7):1364-71.e3.

39. The British Dietetic Association. Model and Process for Nutrition and Dietetic Practice,. 2016, May.

For peer review only

For peer review only - http://bmjopen.bmj.com/site/about/guidelines.xhtml

40. Lara J, Evans EH, O'Brien N, Moynihan PJ, Meyer TD, Adamson AJ, et al. Association of behaviour change techniques with effectiveness of dietary interventions among adults of retirement age: a systematic review and meta-analysis of randomised controlled trials. BMC medicine. 2014;12:177-.
41. Cradock KA, ÓLaighin G, Finucane FM, Gainforth HL, Quinlan LR, Ginis KAM. Behaviour change techniques targeting both diet and physical activity in type 2 diabetes: A systematic review and meta-analysis. International Journal of Behavioral Nutrition and Physical Activity. 2017;14(1):18.
42. Michie S, Richardson M, Johnston M, Abraham C, Francis J, Hardeman W, et al. The behavior change technique taxonomy (v1) of 93 hierarchically clustered techniques: building an international consensus for the reporting of behavior change interventions. Ann Behav Med. 2013;46(1):81-95.
43. Devlin NJ, Brooks R. EQ-5D and the EuroQol Group: Past, Present and Future. Applied Health Economics and Health Policy. 2017;15(2):127-37.
44. Younossi ZM, Stepanova M, Younossi I, Racila A. Validation of Chronic Liver Disease Questionnaire for Nonalcoholic Steatohepatitis in Patients With Biopsy-Proven Nonalcoholic Steatohepatitis. Clin Gastroenterol Hepatol. 2019.
45. Doward L, Balp MM, Twiss J, Slota C, Cryer D, Langford A, et al. Measuring what matters to patients: the development of the NASH-CHECK, a new patient-reported outcome instrument for nonalcoholic steatohepatitis. Journal of Hepatology. 2018;68:S570-S.
46. Martínez-González MA, García-Arellano A, Toledo E, Salas-Salvadó J, Buil-Cosiales P, Corella D, et al. A 14-item Mediterranean diet assessment tool and obesity indexes among high-risk subjects: the PREDIMED trial. PloS one. 2012;7(8):e43134-e.
47. Simpson E, Bradley J, Poliakov I, Jackson D, Olivier P, Adamson AJ, et al. Iterative Development of an Online Dietary Recall Tool: INTAKE24. Nutrients. 2017;9(2).
48. Foster E, Lee C, Imamura F, Hollidge SE, Westgate KL, Venables MC, et al. Validity and reliability of an online self-report 24-h dietary recall method (Intake24): a doubly labelled water study and repeated-measures analysis. J Nutr Sci. 2019;8:e29.
49. Shivappa N, Steck SE, Hurley TG, Hussey JR, Hébert JR. Designing and developing a literature-derived, population-based dietary inflammatory index. Public health nutrition. 2014;17(8):1689-96.
50. Lloyd AJ, Wilson T, Willis ND, Lyons L, Phillips H, Janssen HG, et al. Developing community-based urine sampling methods to deploy biomarker technology for the assessment of dietary exposure. Public Health Nutr. 2020:1-12.
51. Lloyd AJ, Willis ND, Wilson T, Zubair H, Xie L, Chambers E, et al. Developing a Food Exposure and Urine Sampling Strategy for Dietary Exposure Biomarker Validation in Free-Living Individuals. Molecular Nutrition & Food Research. 2019;63(14):1900062.
52. Boyle M, Tiniakos D, Schattenberg JM, Ratziu V, Bugianessi E, Petta S, et al. Performance of the PRO-C3 collagen neo-epitope biomarker in non-alcoholic fatty liver disease. JHEP Reports. 2019;1(3):188-98.
53. Eldridge SM, Chan CL, Campbell MJ, Bond CM, Hopewell S, Thabane L, et al. CONSORT 2010 statement: extension to randomised pilot and feasibility trials. BMJ. 2016;355:i5239.
54. Haigh L, Bremner S, Houghton D, Henderson E, Avery L, Hardy T, et al. Barriers and Facilitators to Mediterranean Diet Adoption by Patients With Nonalcoholic Fatty Liver Disease in Northern Europe. Clin Gastroenterol Hepatol. 2019;17(7):1364-71.e3.
55. Suárez M. Mediterranean Diet and Multi-Ingredient-Based Interventions for the Management of Non-Alcoholic Fatty Liver Disease. Nutrients. 2017;9:1052.
56. Penn L, Boeing H, Boushey CJ, Dragsted LO, Kaput J, Scalbert A, et al. Assessment of dietary intake: NuGO symposium report. Genes Nutr. 2010;5(3):205-13.
57. Younge JO, Kouwenhoven-Pasmooij TA, Freak-Poli R, Roos-Hesselink JW, Hunink MGM. Randomized study designs for lifestyle interventions: a tutorial. International Journal of Epidemiology. 2015;44(6):2006-19.
58. Harris JE, Raynor HA. Crossover Designs in Nutrition and Dietetics Research. Journal of the Academy of Nutrition and Dietetics. 2017;117(7):1023-30.
59. Keogh GF, Cooper GJS, Mulvey TB, McArdle BH, Coles GD, Monro JA, et al. Randomized controlled crossover study of the effect of a highly β-glucan–enriched barley on cardiovascular disease risk factors in mildly hypercholesterolemic men. The American Journal of Clinical Nutrition. 2003;78(4):711-8.
60. Favé G, Beckmann M, Lloyd AJ, Zhou S, Harold G, Lin W, et al. Development and validation of a standardized protocol to monitor human dietary exposure by metabolite fingerprinting of urine samples. Metabolomics. 2011;7(4):469-84.

For peer review only - http://bmjopen.bmj.com/site/about/guidelines.xhtml

61. Logan KJ, Woodside JV, Young IS, McKinley MC, Perkins-Porras L, McKeown PP. Adoption and maintenance of a Mediterranean diet in patients with coronary heart disease from a Northern European population: a pilot randomised trial of different methods of delivering Mediterranean diet advice. Journal of Human Nutrition and Dietetics. 2010;23(1):30-7.

62. Lara J, Turbett E, McKevic A, Rudgard K, Hearth H, Mathers JC. The Mediterranean diet among British older adults: Its understanding, acceptability and the feasibility of a randomised brief intervention with two levels of dietary advice. Maturitas. 2015;82(4):387-93.

63. Berendsen AAM, van de Rest O, Feskens EJM, Santoro A, Ostan R, Pietruszka B, et al. Changes in Dietary Intake and Adherence to the NU-AGE Diet Following a One-Year Dietary Intervention among European Older Adults-Results of the NU-AGE Randomized Trial. Nutrients. 2018;10(12).

64. Downer MK, Gea A, Stampfer M, Sánchez-Tainta A, Corella D, Salas-Salvadó J, et al. Predictors of short- and long-term adherence with a Mediterranean-type diet intervention: the PREDIMED randomized trial. Int J Behav Nutr Phys Act. 2016;13:67-.

Figure legends

Figure 1. Overview of the randomised controlled feasibility trial

Declarations

Authors' contributions

LH, JCM, and QMA conceived of the study; LH, SMcP, JCM, and QMA contributed to design of the study; All authors revised the manuscript for important intellectual content with JCM and QMA giving final approval of the version to be published.

Funding statement

This research was funded by the NIHR Newcastle Biomedical Research Centre (BRC) (grant number BRC-1215-20001). The NIHR Newcastle Biomedical Research Centre (BRC) is a partnership between Newcastle Hospitals NHS Foundation Trust and Newcastle University, funded by the National Institute for Health Research (NIHR). The views expressed are those of the authors and not necessarily those of the NIHR or the Department of Health and Social Care. QMA is a member of the LITMUS (Liver Investigation: Testing Marker Utility in Steatohepatitis) consortium funded by the Innovative Medicines Initiative (IMI2) Program of the European Union (Grant Agreement 777377).

Competing interests statement

The authors have no competing interests to declare that are relevant to the content of this article.

SMcP: Consultancy/speakers fees- Abbvie, Allergan, BMS, Gilead, Intercept, MSD, Novartis and Sequana.

QMA: Research Grant Funding Abbvie, Allergan/Tobira, AstraZeneca, GlaxoSmithKline, Glympse Bio, Novartis Pharma AG, Pfizer Ltd., Vertex. Active Research Collaborations (including research collaborations supported through the EU IMI2 LITMUS Consortium*) Abbvie, Antaros Medical*, Allergan/Tobira*, AstraZeneca*, BMS*, Boehringer Ingelheim International GMBH*, Echosens*, Ellegaard Gottingen Minipigs AS*, Eli Lilly & Company Ltd.*, Exalenz Bioscience Ltd.*, Genfit SA*, Glympse Bio, GlaxoSmithKline, HistoIndex*, Intercept Pharma Europe Ltd.*, iXscient Ltd.*, Nordic Bioscience*, Novartis Pharma AG*, Novo Nordisk A/S*, One Way Liver Genomics SL*, Perspectum Diagnostics*, Pfizer Ltd.*, Resoundant*, Sanofi-Aventis Deutschland GMBH*, SomaLogic Inc.*, Takeda Pharmaceuticals International SA*. Consultancy

For peer review only

For peer review only - http://bmjopen.bmj.com/site/about/guidelines.xhtml

89Bio, Abbott Laboratories, Acuitas Medical, Allergan/Tobira, Altimmune, AstraZeneca, Axcella, Blade, BMS, BNN Cardio, Celgene, Cirius, CymaBay, EcoR1, E3Bio, Eli Lilly & Company Ltd., Galmed, Genentech, Genfit SA, Gilead, Grunthal, HistoIndex, Indalo, Imperial Innovations, Intercept Pharma Europe Ltd., Inventiva, IQVIA, Janssen, Madrigal, MedImmune, Metacrine, NewGene, NGMBio, North Sea Therapeutics, Novartis, Novo Nordisk A/S, Pfizer Ltd., Poxel, ProSciento, Raptor Pharma, Servier, Terns, Viking Therapeutics. Speaker Abbott Laboratories, Allergan/Tobira, BMS, Clinical Care Options, Falk, Fishawack, Genfit SA, Gilead, Integritas Communications, Kenes, MedScape. Royalties Elsevier Ltd (Davidson's Principles & Practice of Medicine textbook)

For peer review only

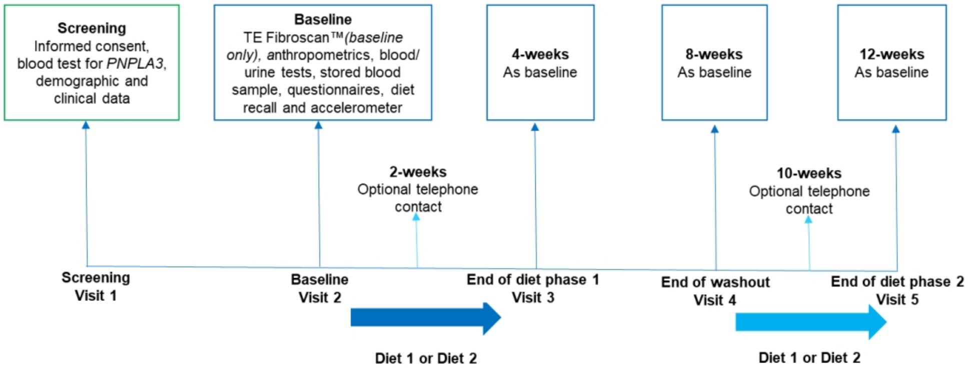

Figure 1. Overview of the randomised controlled feasibility trial

396x162mm (96 x 96 DPI)

Appendix

Table 1. Mediterranean Diet Meal Options

Meal (one serving per container)	Protein(g)	Carb(g)	Fat(g)	Calories
Beef Bolognese with vegetables and whole-wheat pasta	42	43	14	466
Romesco chicken with vegetables and whole-wheat pasta	38	45	17	485
Garlic and herb chicken with vegetable medley	36	27	16	396
Chicken, basil and tomato stir fry	36	40	18	466
Mediterranean falafels with vegetable medley and tahini	10	39	15	331
Cod on vegetable pasta with tomato and basil sauce	30	45	14	426
Halloumi cashew nut and broccoli curry	26	50	23	511
Tomato, black bean with cashews and quinoa	12	36	16	361
Tomato chickpea vegetable curry	14	38	13	353
Chipotle sweet potato chilli with spinach	20	65	6	394
Chicken salad with pesto pasta	36	30	15	399
Garlic and herb chicken salad	32	25	9	309
Tuna and cheese with pesto pasta salad	36	38	12	404
Feta, pine nut and pesto cous salad	16	40	17	377
Red pesto salmon with vegetable medley	24	40	26	490
Honey chilli salmon salad	23	25	20	313
Mozzarella, roasted tomato and pesto pasta salad	19	47	25	477
Grilled halloumi and roasted pepper salad	23	25	23	399
Falafel salad with tahini, avocado and sriracha in Mediterranean herb wrap	20	55	18	462
Chicken with broccoli cashew nut pesto pasta	38	39	17	461
Lentil dahl	23	75	5	437
Vegetable balls and tomato pesto sauce and pasta	12	56	16	416
Chickpea and vegetable curry with rice	23	70	16	516
Butternut, lentil and spinach	15	28	18	372

Appendix 2: Guide for open-response questions

1. How do you feel about the number and duration of the visits and telephone contacts delivered in the trial?

2. What are your thoughts on the main tests we asked you to complete?
   prompts
   - weight, waist, hip and body composition measures
   - blood pressure
   - questionnaires
   - accelerometer
   - urine samples
   - blood samples
   - web-based diet assessment tool

3. Did you complete the main tests and follow the diets as planned? If not, what were the reasons for this?

4. Are there any aspects of the content or delivery of the trial you think we could improve upon?

5. How did your diet and lifestyle patterns change during COVID-19?

For peer review only

The following questions relate to the foods supplied from the Mediterranean diet supplier.

6. Can you tell me your thoughts on the range of foods available?

7. What is your opinion on the taste of the foods that you selected?

8. Can you describe how appealing or unappealing you found the appearance of the selected foods?

9. Can you share your thoughts on how easy, or difficult, was it to use the company's website to order foods?

10. What is your opinion of the delivery times?

11. Do you have any other comments about the Mediterranean meal provider you would like to share with us?

For peer review only

CONSORT 2010 checklist of information to include when reporting a pilot or feasibility trial*

Section/Topic	Item No	Checklist item	Reported on page No
**Title and abstract**			
	1a	Identification as a pilot or feasibility randomised trial in the title	Title page
	1b	Structured summary of pilot trial design, methods, results, and conclusions (for specific guidance see CONSORT abstract extension for pilot trials)	1
**Introduction**			
Background and objectives	2a	Scientific background and explanation of rationale for future definitive trial, and reasons for randomised pilot trial	3
	2b	Specific objectives or research questions for pilot trial	4
**Methods**			
Trial design	3a	Description of pilot trial design (such as parallel, factorial) including allocation ratio	4-5
	3b	Important changes to methods after pilot trial commencement (such as eligibility criteria), with reasons	N/A
Participants	4a	Eligibility criteria for participants	4-5
	4b	Settings and locations where the data were collected	4
	4c	How participants were identified and consented	4
Interventions	5	The interventions for each group with sufficient details to allow replication, including how and when they were actually administered	6-7
Outcomes	6a	Completely defined prespecified assessments or measurements to address each pilot trial objective specified in 2b, including how and when they were assessed	6-8
	6b	Any changes to pilot trial assessments or measurements after the pilot trial commenced, with reasons	N/A
	6c	If applicable, prespecified criteria used to judge whether, or how, to proceed with future definitive trial	9-10
Sample size	7a	Rationale for numbers in the pilot trial	4
	7b	When applicable, explanation of any interim analyses and stopping guidelines	N/A
Randomisation:			
Sequence generation	8a	Method used to generate the random allocation sequence	5
	8b	Type of randomisation(s); details of any restriction (such as blocking and block size)	N/A
Allocation concealment mechanism	9	Mechanism used to implement the random allocation sequence (such as sequentially numbered containers), describing any steps taken to conceal the sequence until interventions were assigned	N/A

For peer review only

Implementation	10	Who generated the random allocation sequence, who enrolled participants, and who assigned participants to interventions	N/A
Blinding	11a	If done, who was blinded after assignment to interventions (for example, participants, care providers, those assessing outcomes) and how	6
	11b	If relevant, description of the similarity of interventions	6-7
Statistical methods	12	Methods used to address each pilot trial objective whether qualitative or quantitative	8-9
**Results**			
Participant flow (a diagram is strongly recommended)	13a	For each group, the numbers of participants who were approached and/or assessed for eligibility, randomly assigned, received intended treatment, and were assessed for each objective	N/A
	13b	For each group, losses and exclusions after randomisation, together with reasons	N/A
Recruitment	14a	Dates defining the periods of recruitment and follow-up	11
	14b	Why the pilot trial ended or was stopped	N/A
Baseline data	15	A table showing baseline demographic and clinical characteristics for each group	N/A
Numbers analysed	16	For each objective, number of participants (denominator) included in each analysis. If relevant, these numbers should be by randomised group	N/A
Outcomes and estimation	17	For each objective, results including expressions of uncertainty (such as 95% confidence interval) for any estimates. If relevant, these results should be by randomised group	N/A
Ancillary analyses	18	Results of any other analyses performed that could be used to inform the future definitive trial	N/A
Harms	19	All important harms or unintended effects in each group (for specific guidance see CONSORT for harms)	N/A
	19a	If relevant, other important unintended consequences	N/A
**Discussion**			
Limitations	20	Pilot trial limitations, addressing sources of potential bias and remaining uncertainty about feasibility	10-11
Generalisability	21	Generalisability (applicability) of pilot trial methods and findings to future definitive trial and other studies	10-11
Interpretation	22	Interpretation consistent with pilot trial objectives and findings, balancing potential benefits and harms, and considering other relevant evidence	N/A
	22a	Implications for progression from pilot to future definitive trial, including any proposed amendments	N/A
**Other information**			
Registration	23	Registration number for pilot trial and name of trial registry	1
Protocol	24	Where the pilot trial protocol can be accessed, if available	N/A
Funding	25	Sources of funding and other support (such as supply of drugs), role of funders	14
	26	Ethical approval or approval by research review committee, confirmed with reference number	11

For peer review only

Citation: Eldridge SM, Chan CL, Campbell MJ, Bond CM, Hopewell S, Thabane L, et al. CONSORT 2010 statement: extension to randomised pilot and feasibility trials. BMJ. 2016;355.

*We strongly recommend reading this statement in conjunction with the CONSORT 2010, extension to randomised pilot and feasibility trials, Explanation and Elaboration for important clarifications on all the items. If relevant, we also recommend reading CONSORT extensions for cluster randomised trials, non-inferiority and equivalence trials, non-pharmacological treatments, herbal interventions, and pragmatic trials. Additional extensions are forthcoming: for those and for up to date references relevant to this checklist, see www.consort-statement.org.

For peer review only
